# Automated laser retraction for targeted glioblastoma coverage during laser interstitial thermal therapy

**DOI:** 10.1002/mp.70267

**Published:** 2026-02-10

**Authors:** Shreeniket Pawar, Yash Sharad Lad, Nageshwar Arepally, Ma'Moun Abu‐Ayyad, Robert Ivkov, Brad E. Zacharia, Constantinos Hadjipanayis, Anilchandra Attaluri

**Affiliations:** ^1^ Department of Mechanical Engineering School of Science, Engineering, and Technology The Pennsylvania State University—Harrisburg Middletown Pennsylvania USA; ^2^ Department of Radiation Oncology and Molecular Radiation Sciences Johns Hopkins University School of Medicine Baltimore Maryland USA; ^3^ Department of Oncology Sydney Kimmel Comprehensive Cancer Center Johns Hopkins University School of Medicine Baltimore Maryland USA; ^4^ Department of Mechanical Engineering Whiting School of Engineering Johns Hopkins University Baltimore Maryland USA; ^5^ Department of Materials Science and Engineering Whiting School of Engineering Johns Hopkins University Baltimore Maryland USA; ^6^ Department of Neurosurgery Pennsylvania State Health Hershey Pennsylvania USA; ^7^ Department of Neurological Surgery University of Pittsburgh School of Medicine Pittsburgh Pennsylvania USA

**Keywords:** fuzzy logic, GBM, MRgLITT, PID, probe retraction

## Abstract

**Background:**

Current magnetic resonance guided laser interstitial thermal therapy (MRgLITT) requires physician input to incrementally retract the laser probe during glioblastoma (GBM) treatment, to achieve maximum safe lesion coverage (LC).

**Objective:**

Through this computational study, we propose an automated MRgLITT system based on temperature and thermal damage feedback to enable precise laser probe positioning and achieve the target LC during GBM treatment.

**Methods:**

Our goal was to design a cascaded proportional‐integral‐derivative (PID) controller with a fuzzy logic controller to achieve thermal damage, Ω ≥ 0.99 within 90% of the target tumor volume. Two modelling approaches were explored for PID control system to maintain the tumor boundary temperature at: (i) constant optical properties and (ii) variable optical properties. A fuzzy‐logic controller was used to incrementally retract the laser probe once the thermal damage at the corresponding boundary setpoint reached the target (Ω = 0.99). The PID and fuzzy logic controller was designed in the system dynamic modeling software, MATLAB Simulink, and bioheat transfer physics was modelled using the finite element analysis (FEA) software, COMSOL Multiphysics. FEA simulations were performed on a de‐identified patient voxel model consisting of domains such as the skull, CSF, brain and tumor. The laser probe was modeled as a cylinder with a diameter of 1.65 mm and tip length of 5 mm. The temperature and thermal damage measuring probes were modeled to represent the thermal imaging planes from clinically approved LITT systems.

**Results:**

The results indicated that the automated control framework maintained the tumor boundary temperature at 60°C. The designed automated control framework did not achieve the target LC but increased the LC by ∼25% with reduction in treatment time.

**Conclusion:**

The results of this computational study indicate that the designed automated MRgLITT approach has potential to improve the LC, reduce treatment time and operator specific variability. Future efforts will focus on developing and validating the proposed approach.

## 1 INTRODUCTION

Magnetic resonance guided laser interstitial thermal therapy^1^, ^2^ (MRgLITT) is a minimally invasive stereotactic thermal ablation technique, commonly used to treat recurrent and newly diagnosed glioblastoma (GBM). MRgLITT offers a favorable treatment option than surgery for recurrent GBM^2^ (rGBM). It effectively addresses challenges related to the diffuse nature and location^3^ of recurrence. Additionally, it overcomes tumor resistance to therapies^4^ and accommodates patients with lower performance status who may not qualify for open surgery. In such cases, MRgLITT serves as a suitable treatment option due to its minimally invasive nature. Reported studies indicate that MRgLITT induces a hyperthermic rim^5^, ^6^ (∼1–2 cm) beyond the ablative zones. Temporary disruption of blood‐brain barrier (BBB) is observed in the peritumoral hyperthermic rim. Disruption of BBB,^7^, ^8^ post MRgLITT provides an opportunity to combine other therapies such as chemotherapy or immunotherapy to target the infiltrative tumor cells reducing likelihood of recurrence.

During MRgLITT, with the help of neuronavigation, a laser catheter is carefully inserted into the lesion through a small opening in the skull.^9^, ^10^ Physician applies a short laser pulse to confirm the probe location on with the help of magnetic resonance temperature imaging (MRTi) guidance. MRTi enables real‐time generation of temperature and thermal damage maps during the procedure, allowing control of laser energy by the physician. Later the laser energy (power, exposure time, position) is adjusted based on the real time MRTi feedback (thermal map and damage contour). Once that location is treated, physician manually initiates the incremental laser retraction to cover additional lesion volume. Two distinct types of fibers are available for MRgLITT: a full‐fire (Medtronic Visualase^11^ and Monteris NeuroBlate^12^) that allows for three‐dimensional (3D) radial thermal damage and a side‐fire (Monteris NeuroBlate) that permits thermal damage conformity in complex geometry. However, this manual process^13^ of pulsed laser application and incremental laser retraction is labor‐intensive and requires a significant learning curve^14^ to achieve consistent lesion coverage (LC, percentage of the tumor with thermal damage exceeding 0.99). Therefore, over a period, physicians started using lower lesion volume as selection criteria for MRgLITT.^14^

Achieving target LC requires maintaining an ablation temperature of at least 60°C^15^ at the tumor boundary for a minimum duration of 10 min,^16^ while minimizing damage to surrounding healthy tissue. Intralesional temperatures exceeding 100°C^9^ can cause vaporization around the laser probe, leading to air bubble formation. This results in charring, carbonization, and tissue shrinkage.^9^ These effects limit laser penetration into the tissue and produce inaccurate temperature readings on MRTi, ultimately reducing LC.

Prior studies have suggested the design of control systems^17^, ^18^ for MRgLITT. However, the controllers do not include thermal damage control during MRgLITT treatments. Additionally, these models do not account for incremental retraction of the laser probe during the treatment. Controller design software such as MATLAB Simulink^19^ allows for the design of linear temporal models but lacks the capability to accurately represent the spatiotemporal dynamics of the system which will lead to suboptimal treatment plan. Finite element bioheat transfer (FEBHT) can effectively model these spatiotemporal dynamics. However, FEBHT^20^ is not well‐suited for solving multi constraint control problems. Integrating controller design software with FEBHT^21^ in real time offers a way to design and simulate control systems. This combined approach offers accurate way to design in‐silico control systems for MRgLITT.

The objective of this study was to develop a fully automated treatment framework for MRgLITT that enables real‐time control of thermal damage delivery and automated probe retraction based on thermal damage feedback. The proposed system is designed to maintain temperatures at the tumor boundary within the therapeutic ablation range (∼60°C), while limiting the maximum treatment temperature to below 100°C. By leveraging real‐time voxel‐based MRTi feedback, the system dynamically adjusts laser power to ensure precise and safe ablation. This approach minimizes dependence on operator expertise, promotes consistent treatment outcomes, and reduces procedural risks in current clinical MRgLITT. Automated probe retraction further enhances LC while protecting adjacent healthy tissue. Collectively, these advancements improve workflow efficiency, treatment safety, and patient outcomes by enabling more effective and reproducible therapy. The benefits of this framework may be further augmented using a side‐fire laser probe, which can accommodate irregular tumor geometries and address asymmetric heating effects caused by surrounding fluidic structures. To achieve this a deidentified human head anatomy from a previous study^22^ was segmented and imported into the FEBHT software, COMSOL Multiphysics^23^ (Burlington, MA, USA). A full‐fire laser catheter was modeled in FEBHT software based on the specifications of the Medtronic Visualase MRgLITT system.^11^ A proportional‐integral‐derivative^24^ (PID) controller was designed to maintain the target temperature within the tumor, as determined by the user. Additionally, a fuzzy‐logic controller^25^ was designed for the incremental retraction of the laser probe based on thermal damage feedback. Simulation results indicate that the cascaded control framework increased the LC by maintaining tumor boundary temperature at 60°C. The increased LC was achieved with minimal damage to the surrounding healthy tissue. Also, the controlled MRgLITT framework reduced the treatment time by, compared to the reported clinical time. These findings suggest that an automated system could improve LC in clinical MRgLITT procedures.

## 2 MATERIALS AND METHODS

**FIGURE 1:**
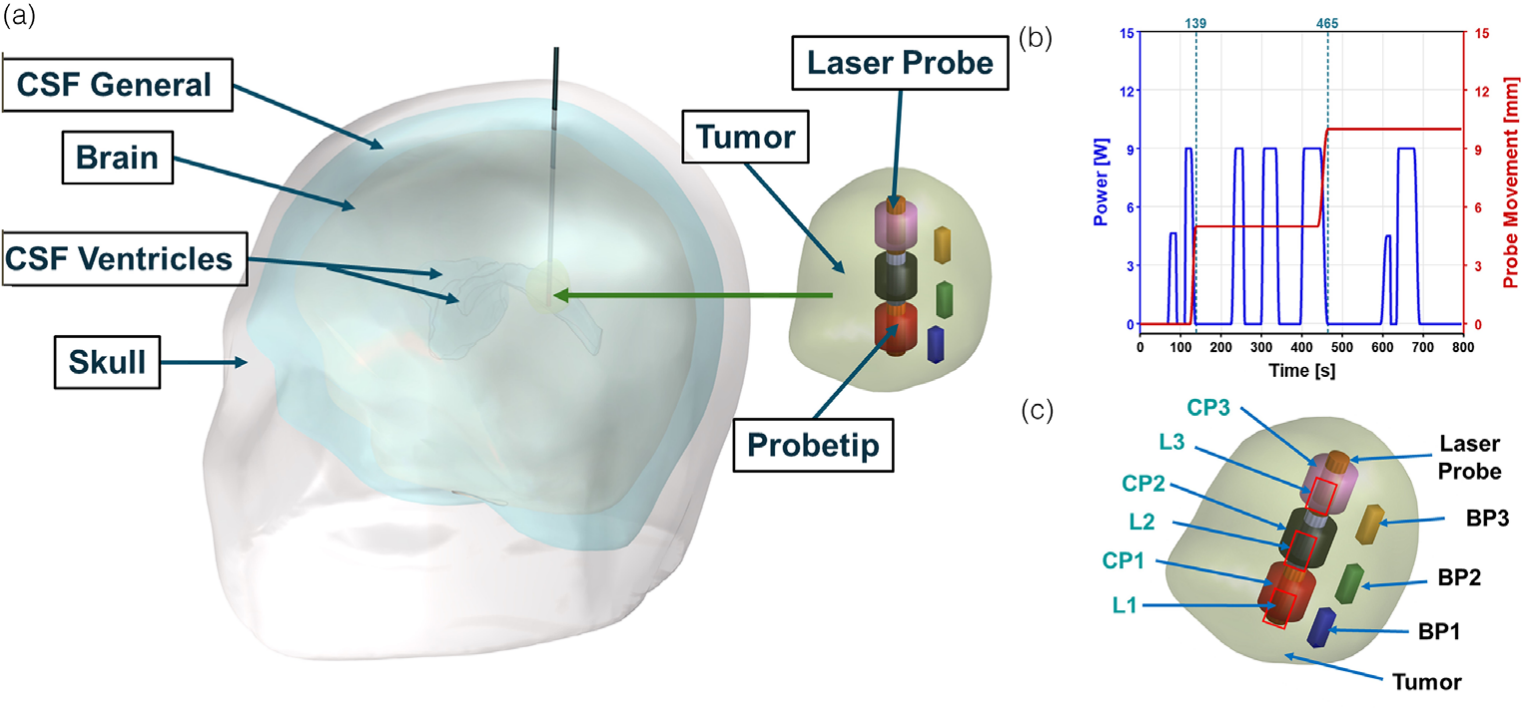
Geometry and laser log obtained from de‐identified patient dataset, demonstrating probe placement for controller feedback. (a) Three‐dimensional human head geometry, obtained from a de‐identified magnetic resonance guided laser interstitial thermal therapy (MRgLITT) patient, was segmented from MR images. This geometry was partitioned into five distinct domains, representing the skull, cerebrospinal fluid (CSF) general, brain tissue (averaged white matter and gray matter), CSF ventricles, and the tumor region along with MNP distribution. (b) The laser power and incremental laser retraction (5 mm) were modeled using a laser log from the de‐identified MRgLITT treatment. (c) Placement of temperature and thermal damage measuring probes within the tumor to provide feedback to the controller. L1, L2, and L3 represent the incremental laser retraction. CP1, CP2 and CP3 represent the maximum tumor temperature, whereas BP1, BP2, and BP3 represent the tumor boundary temperature.

A deidentified human head model used in this study was repurposed from our previous work,^22^ as shown in Figure 1a. This dataset was used as a benchmark to compare the performance of our proposed control strategy against the clinical LITT scenario in which we used LC as the metric to compare current LITT outcome to the proposed control system outcome. Briefly, MRI images obtained from de‐identified human anatomy, who underwent MRgLITT, were segmented using commercial software Materialize MIMICS into different domains. The domains considered were the skull, cerebrospinal fluid (CSF) general, brain (averaged white matter and grey matter), CSF ventricles, and tumor. The segmented geometry was imported into the commercial FEBHT software, COMSOL Multiphysics.

To perform the bioheat transfer calculations, we used a modified form of Pennes’ time‐dependent partial differential equation,^26^ represented by Equation (1) which we verified^27^ and validated^28^ using MRgLITT data (refer Supplementary information). This equation considers factors such as blood perfusion, heat source owing to laser tissue interaction, and tissue metabolic heat rates. Equation (2) represents the heat sink due to microvascular blood perfusion in different domains.

(1) $$\rho_{i}C_{p,i}\;\frac{\partial T}{\partial t}=\nabla \left(k_{i}\nabla T\right)+Q_{\mathrm{perf},i}+Q_{\mathrm{Laser}}+Q_{\mathrm{met},i}$$

(2) $$\;Q_{\mathrm{Perf},i}=\rho_{b}C_{p,b}\omega_{b,i}\left(T_{b}-T\right)$$

In Equations (1) and (2), ρ is the density kg· m^−3^, *C_p_* is the specific heat capacity J(kg K)^−1^, *k* is the thermal conductivity W(m· K)^−1^, $\rho_{b}$ is the blood density kg m^−3^, *C_p,b_* is the specific heat capacity of blood J(kg·K)^−1^, $\omega_{b}$ is the microvascular blood perfusion s^−1^, $Q_{\mathrm{perf},i}$ is the heat sink due to microvascular blood perfusion W m^−3^, $Q_{\mathrm{Laser}}$ is the laser heat source W m^−3^and $Q_{\mathrm{met},i}$ is the metabolic heat rate W m^−3^. Subscript ‘*i*’ denotes the specific (tissue) domain under consideration, that is, skull/CSF/brain/tumor. Once the governing bioheat transfer equation was modeled within the FEA software, appropriate material properties^29^, ^30^, ^31^, ^32^, ^33^ were assigned to different domains, as shown in Tables S1 and S2.

The modeling of CSF (general and ventricles) and perfusion followed the same approach used in our previous study.^22^ Briefly, the CSF domains were modeled using convectively enhanced conductivity^34^, ^35^, ^36^, ^37^ approach within the FEBHT software. Thermal damage was modeled using Arrhenius first order kinetics.^38^ Thermal damage dependent blood perfusion^39^, ^40^, ^41^, ^42^, ^43^, ^44^ was modeled within the tumor and peritumoral domains, (refer to Supplementary information).

### 2.1 Modeling laser heat source

The laser wavelength used in our simulations was 980 nm assuming the Medtronic Visualase MRI‐Guided Laser Ablation system.^11^ This wavelength was selected since the deidentified patient dataset used as a benchmark in this study was treated using Medtronic Visualase system. We compared two ways to model the laser: (i) Point Source Laser Heating (PSLH)^45^, ^46^ (ii) Distributed point heat sources (DPHS) in the laser tip^47^ (Refer supplementary information for more information).

### 2.2 Temperature dependent thermal properties

Thermal properties of the brain and tumor were modeled using temperature‐dependent exponential expressions.^48^ In contrast, the skull and CSF were assumed to have constant thermal properties. This choice was based on the observation that thermal conductivity in these regions increases significantly only above 50°C, while the simulated temperatures remained below 40°C. As a result, temperature dependence in the skull and CSF was considered negligible. To reduce computational complexity without compromising accuracy, we did not apply temperature‐dependent properties in these regions.

### 2.3 Thermal damage dependent optical properties

We compared two approaches to model the dynamic thermal damage dependent optical properties: (i) Multi‐optical parameter^49^ and (ii) Single optical parameter.^50^ Mulit‐optical parameter method provides the most accurate representation of changes in optical properties due to thermal damage. However, it increases computational complexity by expanding the parameter space to three variables during treatment. Single optical parameter model reduces the parameter space to a single variable, $\mu_{eff}$, making it more computationally efficient while still capturing the essential trend of optical property changes with thermal damage. If this approach predicts $\mu_{eff}$ within 5% then the single optical parameter for modelling would be used. Optical properties^51^ are used in the study are shown in Table S3 (Refer Supplementary information for more information).

### 2.4 Modeling incremental retraction of the laser probe from a de‐identified MRgLITT laser log

In this study, the laser power was modeled based on the laser log obtained from de‐identified MRgLITT treatment. This laser log was modeled in FEA software using a piecewise linear function, as shown in Figure 1b, defining the ON and OFF intervals of the laser power. In addition, the retraction of the laser probe was modeled as a dynamically moving heat source using a similar piecewise linear function. Figure 1b shows the movement of the heat source (5 mm increments) in accordance with the laser log.

### 2.5 MRgLITT temperature and thermal damage probes

During MRgLITT, temperature is monitored across three imaging planes, with each voxel measuring 1 mm × 1 mm × 3 mm. For modeling purposes, we assumed that we have temperature and thermal damage at six voxel positions three near the tip and three at the boundary as shown in Figure 1c.

The initial center and boundary probes were denoted as CP1 and BP1, respectively. We assumed that the MRTi provides the average temperature and thermal damage values within each voxel. As the probe is retracted, new probe locations were considered and labeled as CP2, CP3 (center probes) and BP2, BP3 (boundary probes), respectively. It was assumed that the CP1 BP1 pair, CP2 BP2 pair and CP3 BP3 pair were in the same plane.

For the center probe, we modeled the voxel as a cylindrical tube due to the symmetry of the heat source and the distance from the tumor boundary. The inner diameter of the tube was taken as 1.65 mm, corresponding to the laser tip diameter, with a wall thickness of 1 mm (equal to the voxel dimension). The height of the cylinder was set to 3 mm, matching the voxel height.

The boundary voxel was modeled as a cuboid with dimensions of 1 mm × 1 mm × 3 mm. One face of this cuboid was assumed to touch the tumor boundary, while the entire volume remained within the tumor region.

### 2.6 Initial and boundary conditions

Initially, all regions were assumed to be at baseline temperature of 37°C, corresponding to normal body temperature. A convective boundary condition was applied to the outer surface of the skull, with a convective heat transfer coefficient^52^ set at 5 W (m^2^ K)^−1^, and an ambient temperature of 20°C. Also, to simulate the catheter cooling a Robbins or boundary condition of third kind was applied between the laser probe and tumor with a convective heat transfer coefficient^53^ set at 100 W (m^2^ K)^−1^, and an ambient temperature of 20°C.

### 2.7 Discretization, numerical scheme and solver settings

The Pennes bioheat equation was discretized using a quadratic Lagrange function combined with a second‐order backward difference formula, employing a fixed time step of 1 s. An implicit time‐stepping scheme was implemented, and a parallel sparse direct solver was used to solve the equations.

### 2.8 Optical properties uncertainty quantification and propagation

To quantify the uncertainty in native and coagulated optical properties, we used the sensitivity analysis feature within the Uncertainty Quantification module of COMSOL Multiphysics. Specifically, we applied the SOBOL variance‐based method, which decomposes output variance into contributions from each input parameter and their interactions, making it well suited to capture nonlinear effects in thermal models. The quantities of interest included the thermal damage at BP1, BP2, and BP3. A surrogate model was constructed using an adaptive sparse Polynomial Chaos Expansion (PCE) with a relative tolerance of 0.001, and its accuracy was validated against 10 verification points. A total of 20 samples were used to generate the PCE surrogate, with all uncertain parameters assumed independent. Convergence was verified by doubling the sample size, which produced changes in SOBOL indices of less than 0.02, and reproducibility was ensured by fixing the random seed. The resulting uncertainty estimates were reported using the 10^th^ to 90^th^ percentile confidence interval.

### 2.9 Designing a cascaded PID fuzzy logic control system

The tumor boundary and maximum tumor temperatures were controlled within ablative limits using a PID controller.^24^ Equations (3) and (4) show the governing equation for the PID controller.

(3) $$U_{\mathrm{ctrl}}=K_{p}\;\cdot e\left(t\right)+K_{i}\cdot \int_{0}^{t}e\left(\tau \right)\;d\tau +K_{d}\cdot \frac{d\;e\left(t\right)}{\mathrm{dt}}$$

(4) $$P_{\mathrm{Laser}}\;\left(T\right)=U_{\mathrm{ctrl}}\;\times P_{\max}$$

where $K_{p}\;W\cdot {\left(K\right)}^{-1}$ is the proportional gain, $e\left(t\right)\;K\;=T_{SP}\;-T_{P_{i}}$ is the error (i.e., the difference between the temperature setpoint and the control temperature of the *i^th^* probe), $K_{i\;}W\cdot {\left(K\cdot s\right)}^{-1}$ is the integral gain, and $K_{d}\;W\cdot s\cdot {\left(K\right)}^{-1}$ is the derivative gain. PID gains were tuned manually using trial and error method owing to highly non‐linear system dynamics. The gains used for the study are listed in Table 1. Initially, the proportional gain $K_{p}$​ was varied while setting the integral and derivative gains ($K_{i}$ and $K_{d}$) to zero. Adjustments to $K_{p}$​ were made until the temperature response closely approached the setpoint. Once further changes in $K_{p}$ produced negligible improvements in the temperature response, the value was fixed. Subsequently, $K_{i}$​ was tuned by varying it while keeping $K_{p}$​ constant and $K_{d}$​ set to zero. Finally, $K_{d}$ was adjusted with $K_{p}$​ and $K_{i}$​ held constant.

**TABLE 1:** Parameters for PID controller.

Description	Value
K_p_ W/(°C)	7.5
K_i_ W/(s °C)	0.5
K_d_ W s/°C	0
Setpoint temperature at CP1°C	100
Setpoint temperature at CP2°C	100
Setpoint temperature at CP3°C	100
Setpoint temperature at BP1°C	60
Setpoint temperature at BP2°C	60
Setpoint temperature at BP3°C	60
Setpoint thermal damage at BP1	0.99
Setpoint thermal damage at BP2	0.99
Setpoint thermal damage at BP3	0.99

**FIGURE 2:**
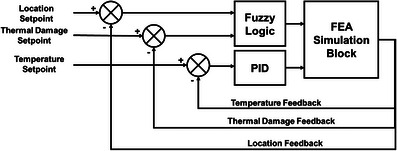
Block diagram of cascaded proportional integral derivative (PID) fuzzy logic controller for thermal damage control during MRgLITT treatments.

Retraction of the laser probe was achieved by a fuzzy‐logic controller using feedback from the thermal damage for which temperature is obtained from the measuring probes. The cascaded controller was designed in MATLAB Simulink,^19^, ^21^ with temperatures measured at the tumor center and tumor boundary as inputs. The laser power output (Equation 3) from the controller was applied to the co‐simulation block, which represented the dynamic finite element simulations of the system. The controller was given feedback on the maximum temperature of the laser tip, as well as boundary probes CP1, CP2, CP3, BP1, BP2, and BP3. Clinical MRgLITT aims to prevent temperatures near 100°C to avoid charring and vaporization.^9^ Therefore, center temperatures (CP1–CP3) were constrained to 100°C. Additionally, the temperature at the boundary probes (BP1–BP3) should maintained within the ablative zone of 50°C–60°C.^15^ Therefore, to minimize treatment time, the boundary probe (BP1–BP3) temperatures were constrained to 60°C. In addition, at the boundary probes thermal damage was evaluated from the temperature, which served as feedback to the fuzzy logic controller to achieve laser probe retraction. The laser probe was pulled back by 5 mm each when the thermal damage at boundary probes 1, and 2 reached Arrhenius damage of 0.99. The controller output was set to zero when the evaluated thermal damage at any boundary probe reached the thermal damage value of 0.99 for all three probes. Figure 2 shows a block diagram of the cascaded PID‐Fuzzy Logic controller.

## 3 RESULTS

### 3.1 Verification and validation

Figure S3 (Supplementary information) present the verification of the Pennes' bioheat transfer equation and and Figures S4 and S5 (Supplementry Information) present validation of the Pennes’ bioheat transfer equation and PID controller, respectively. The verification results show that the analytical and numerical solutions at multiple radial positions agree within 0.1%, confirming the effectiveness of the solver, mesh resolution, and time step used in this study. Validation results with the MRgLITT system demonstrate that predicted temperatures from at least one of our perfusion values (low or high) are within 10% of MRTi measurements. Furthermore, the results fall between the MRTi data and the high‐fidelity FEA model by Fuentes et al.,^28^ which incorporates voxel‐wise thermal properties and perfusion, indicating strong agreement and predictive capability of our model.

### 3.2 Comparison of optical parameter and laser heat source modelling

Figure S6 (Supplementary information) shows the effect of multiple and single optical parameters. We observed that the maximum relative difference was ∼1% which occurs at thermal damage of 0.5. Since the maximum difference was ∼1% for modeling damage dependent (variable optical) properties we used a single optical parameter approach. Next, we compared two laser heat source modelling approaches at constant optical properties, and our results indicate that PSLH and DPHS in the tip yield similar thermal damage and temperature which are within 5% as shown in Figure S7 (Supplementary information) and Table S9. This is due to comparable voxel and laser tip size. Hence, we modeled laser using PSLH in further study.

### 3.3 Comparison of modelling constant and variable optical properties

**FIGURE 3:**
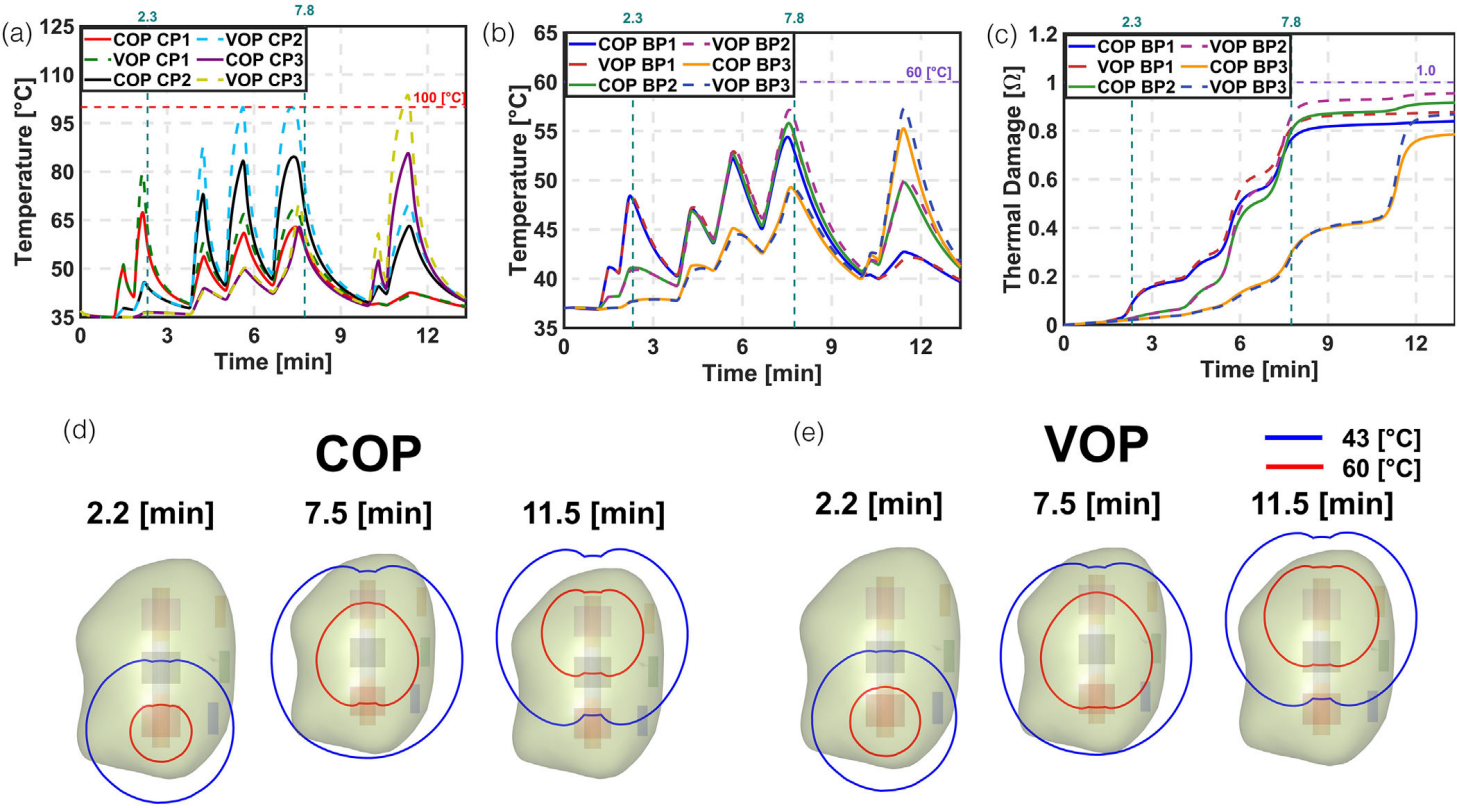
Comparison of temperature and thermal damage at CP and BP for modelling constant and variable optical properties (COP and VOP). (a) Temperature at CP. (b) Temperature at BP. (c) Thermal damage at BP. (d) Temperature contours of 43°C and 60°C at the maximum temperature reached during L1, L2, and L3 for COP. (e) Temperature contours of 43°C and 60°C at the maximum temperature reached during L1, L2, and L3 for VOP.

Figure 3 compares the modeling results of the COP and VOP approaches. During the initial pulse, temperature and thermal damage showed minimal variation. This indicates that no significant thermal damage occurred. In later pulses, the temperature at the CP increased due to reduced optical penetration depth. However, the temperature and thermal damage at the BP remained nearly the same for both COP and VOP models, suggesting similar optical properties. To ensure consistent deposited power across COP and VOP, we applied a constant scaling factor (Equation S7). Despite this, the actual deposited power differed by up to 15%, with the VOP approach showing slightly higher values. In the final two pulses, both temperature and thermal damage increased slightly under the VOP model, reflecting the effect of updated optical properties. We used nominal values for thermal damage in this analysis. If the effective attenuation coefficient (µ_eff_) were significantly higher, damage at the BP might not be observed. The LC at the end of treatment was ∼32% for COP and 41% for VOP, indicating that over 50% of the tumor remained untreated.

### 3.4 Parametric sweep on attenuation co‐efficient

**FIGURE 4:**
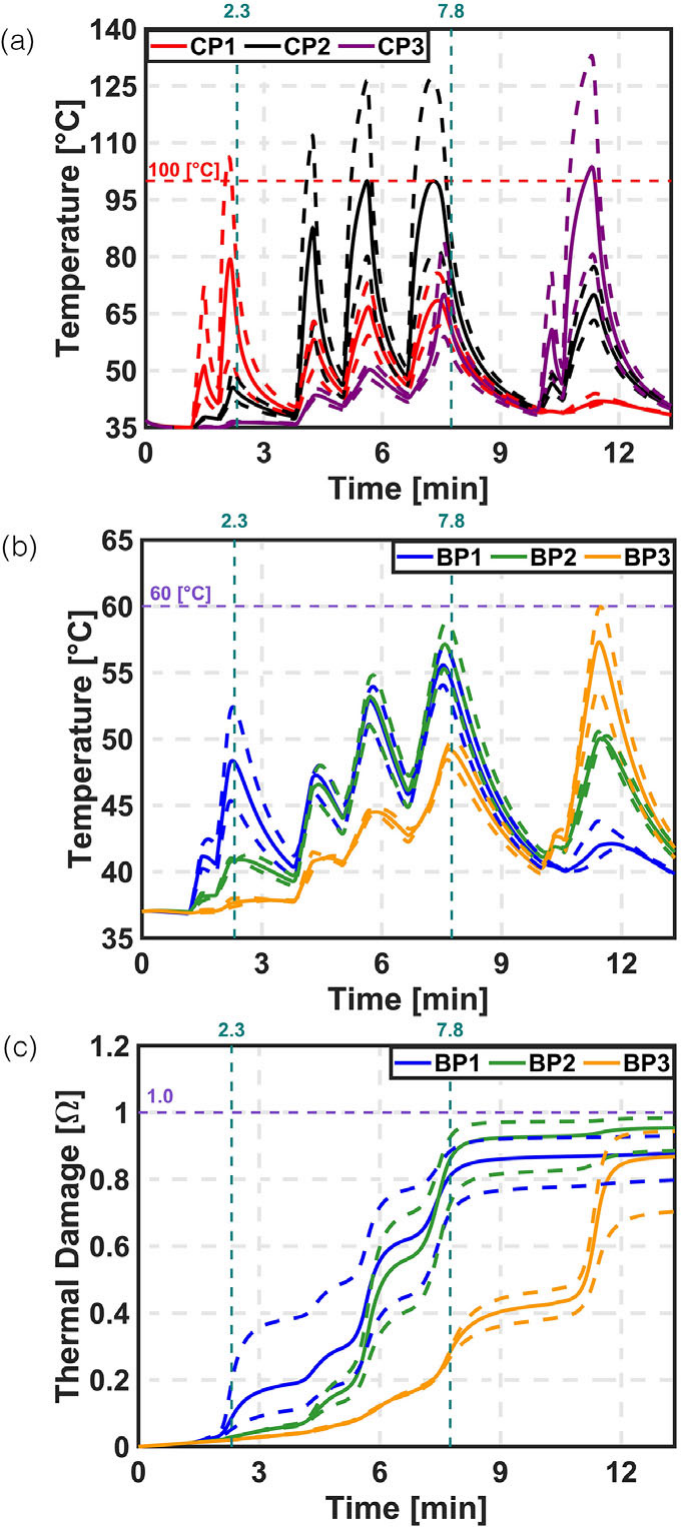
Parametric sweep on attenuation co‐efficient for VOP. Comparison between temperature and thermal damage at CP and BP. (a) Temperature at CP. (b) Temperature at BP. (c) Thermal damage at BP.

Figure S8 (Supplementary information) and Figure 4 show the effect of varying the attenuation coefficient for both COP and VOP approaches. The results indicate that temperature and thermal damage varied significantly with changes in the attenuation coefficient. At the end of treatment, thermal damage at boundary points BP1, BP2, and BP3 ranged from approximately 70%–91%, 78%–97%, and 62%–90% for COP, and from 79%–92%, 88%–98%, and 70%–94% for VOP, respectively. These variations reflect a substantial impact on treatment outcomes. Furthermore, the LC at the end of treatment ranged from 13%–46% for COP and 27%–51% for VOP.

### 3.5 Uncertainty quantification and propagation

**FIGURE 5:**
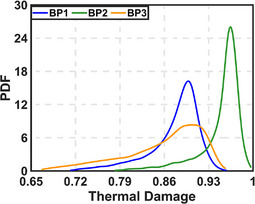
Probability distribution function for thermal damage by varying the attenuation co‐efficient at three BPs.

Table 2 presents the uncertainty quantification (UQ) for thermal damage at the BP locations. Among all input parameters, the coagulated attenuation coefficient contributes the most to the overall uncertainty. The interaction effects remain below 10%, indicating a predominantly linear relationship in the quantified uncertainty. When this uncertainty is propagated to thermal damage estimates at the BPs, BP3 shows the widest range, while BP2 exhibits the narrowest. The probability distributions of thermal damage at all BPs are right‐skewed (mode greater than median), suggesting that although the most likely estimate is high, lower damage outcomes are more frequent (Figure 5).

**TABLE 2:** Total uncertainty quantification (UQ) using SOBOL method for thermal damage at BP with attenuation co‐efficient (native and coagulated) as uncertainty parameter.

Description		Thermal damage		
		BP1	BP2	BP3
First order UQ	$\mu_{\mathrm{eff},n}$	0.08	0.04	0.02
	$\mu_{\mathrm{eff},c}$	0.86	0.94	0.97
Total UQ	$\mu_{\mathrm{eff},n}$	0.14	0.06	0.03
	$\mu_{\mathrm{eff},c}$	0.92	0.96	0.98

Figure S9 (Supplementary information) shows that the logarithm of the survival probability (1 − Ω) approximates a Gaussian distribution at the most likely thermal damage estimate for each BP.

Table 3 shows the propagated uncertainty during the simulations.

**TABLE 3:** Propagated uncertainty for metrics thermal damage due to attenuation co‐efficient (native and coagulated) at BPs.

Description	Thermal damage		
	BP1	BP2	BP3
Mean	0.88	0.95	0.86
Standard deviation	0.04	0.03	0.06
Minimum	0.71	0.78	0.67
Maximum	0.96	0.99	0.96
Lower 95%	0.76	0.84	0.71
Higher 95%	0.93	0.98	0.94
Model error	0.01	0.02	0.01

### 3.6 PID controller

#### 3.6.1 Constant optical properties (COP) approach

**FIGURE 6:**
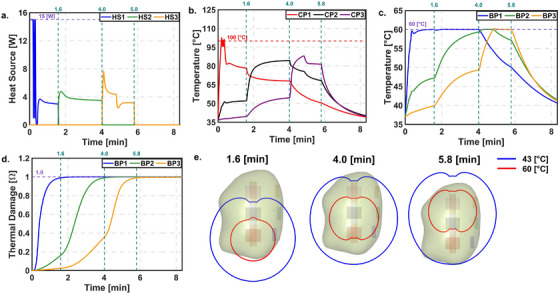
PID controller with automatic probe retraction for COP. (a) Power. (b) Temperature at CP. (c) Temperature at BP. (d) Thermal damage at BP. (e) Temperature contour of 60 and 43°C at the end of L1, L2, and L3 respectively.

Figure 6 shows the results of the PID controller during laser probe retraction using the COP approach. Initially, the temperature at CP1 reached 100°C, while BP1 remained below 60°C. As a result, the HS1 turned ON and OFF due to the safety constraint. The target temperature at BP1 was achieved at ∼0.33 min, and the target thermal damage of 0.99 was reached at 1.6 min. Following this, the probe was retracted and heat source HS2 was activated. During this phase, the temperature at BP1 remained higher than at BP2. To avoid exceeding the 60°C limit at any boundary point, the controller used BP1 as the reference for temperature regulation. The target thermal damage of 0.99 was reached at BP2 at 4.0 min. The probe was then retracted again, and HS3 was activated to control the temperature based on BP3. The target thermal damage at BP3 was reached at 5.8 min, concluding the treatment. The LC at the end of the treatment was 76.51%.

#### 3.6.2 Variable optical properties (VOP) approach

**FIGURE 7:**
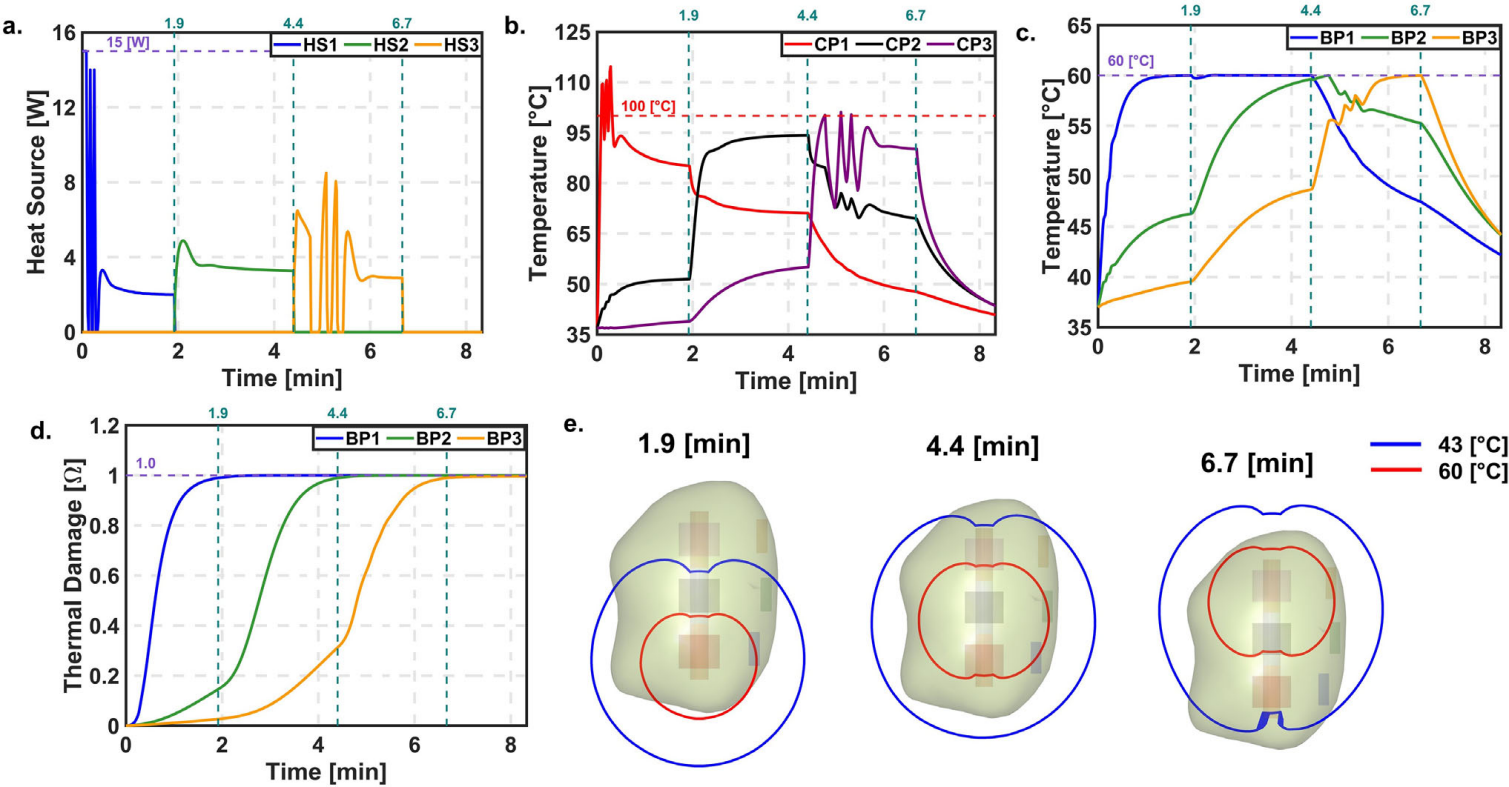
PID controller with automatic probe retraction for VOP. (a) Power. (b) Temperature at CP. (c) Temperature at BP. (d) Thermal damage at BP. (e) Temperature contour of 60 and 43°C at the end of L1, L2, and L3 respectively.

Figure 7 presents the results of the PID controller during laser probe retraction using the VOP approach. The overall behavior was similar to the COP approach; however, more temperature oscillations and a longer time to reach steady state were observed in HS1, especially when CP1 exceeded 100°C. The target thermal damage at BP1 was reached at 1.9 min. As in the COP case, temperature control was based on BP1, and the target thermal damage was achieved at 4.4 min, after which HS3 was turned ON. In contrast to the COP results, CP3 reached 100°C in HS3, causing the controller to switch ON and OFF intermittently. This led to a delay in achieving the target thermal damage at BP3, which was finally reached at 6.7 min. Although the treatment time increased compared to the COP approach, the LC remained approximately the same at 74.61%. Performance metrics for COP and VOP are shown in Table S11.

## 4 DISCUSSION

MRgLITT is a clinically approved therapy for treating rGBM^1^, ^2^ and is being explored for newly diagnosed GBM. Previous studies show that LC depends on operator learning curve^14^ and improves after significant number (∼20–25) of treatments with some amount of automation during MRgLITT. In this study, we demonstrated the feasibility of a fully automated thermal damage feedback control system for MRgLITT to minimize prolonged learning curve, reduce operator dependency, improve LC and reduce treatment time. We modeled clinical pulsed heating scenario with temperature dependent thermal properties of MRgLITT from a de‐identified patient data with two approaches in commercial FEBHT software.^21^

The first approach used PSLH, an approximate method to model laser heating. The second approach used DPHS, where the heat source was distributed along the entire laser tip. A comparison of both methods showed similar heating and thermal damage at the boundary point (BP), with differences within 5%. This is because the observed voxel had a height of 3 mm, matching the laser tip length. As a result, the DPHS method closely approximated PSLH without significant loss of detail. However, if MRTi voxel resolution improves in the future, DPHS may become necessary for accurate treatment planning.

Next, we studied the impact of damage‐dependent optical properties by increasing the attenuation coefficient by a factor of 1.5. This increase aligns with reported values in brain tissue, where attenuation typically rises by 1.5–2.5 times.^51^ We found that tissue coagulation raised center temperatures by ∼5°C–10°C. However, these changes in optical properties also altered the power deposited in tissue. To address this, we introduced a scaling factor to maintain consistent power deposition. Even with this adjustment, the deposited power varied within 10%–15%, highlighting the need for more robust methods to account for damage‐dependent optical changes while conserving deposited power.

We also examined the effect of uncertainty in physical parameters on treatment outcomes. Using screening from our previous study,^54^ we found that thermal properties had limited impact, while optical properties played a key role in determining treatment outcomes. Therefore, we conducted uncertainty quantification using the SOBOL method, which relies on variance‐based analysis. The results showed that coagulated tissue properties had the most influence on thermal damage. This suggests that during a significant portion of treatment, optical properties evolve with tissue damage. SOBOL indices further indicated that the covariance between native and coagulated tissue remained below 10%, implying that a linear response surface model could be sufficient.

Uncertainty quantification also showed that thermal damage at BP2 could be predicted with high confidence, while confidence in predictions for BP3 was lower. This may be due to the exponential nature of thermal damage accumulation, which makes it difficult to quantify beyond ∼0.95, as the damage curve begins to saturate. This reinforces the need for uncertainty quantification and supports the use of an automated controller to reduce operator dependency.

Comparison of our simulations with previous studies (Figures S4^28^ and S5^55^) showed that predicted temperatures were within 10% of MRTi data. Our FEA model assumed isotropic thermal and physiological properties, unlike Fuentes et al.^28^ Optimizing for node‐specific heterogeneity would require significantly greater computational power. As shown in Figure S5, an alternative strategy^55^ for addressing non‐homogeneous properties is to employ a controller capable of compensating for these variations. Specifically, constant power delivery (Figure S5a) resulted in a discrepancy of ∼1.5°C, likely due to incomplete knowledge such as voxel position or heterogeneous tissue properties. In contrast, introducing the controller (Figure S5b and S5c) reduced this discrepancy, bringing performance within the standard deviation reported by Desclides et al.^55^ These findings suggest that automated control not only mitigates the impact of tissue heterogeneity but may also compensate for uncertainties in voxel positioning.

To address this, we designed a fully automated thermal damage feedback control system for MRgLITT. This system aims to reduce the learning curve, minimize operator input, and improve confidence in treatment. Results showed that the automated controller achieved the target LC with both COP and VOP, while also reducing treatment time compared to the clinical pulsed heating method.

COP and VOP methods yielded similar LC values. The controller effectively compensated for variations in tissue properties, as the thermal damage model itself does not rely on those properties. While heterogeneity is managed by the controller, the damage response still depends on a separate parameter activation energy which we held constant. Future studies should investigate the impact of varying this parameter. Additionally, the selection of voxels for the PID controller was done arbitrarily, and future work should explore optimized strategies for voxel selection.

Another important observation came during the transition from heat source HS1 to HS2. Using the current state‐of‐the‐art pulsing method, BP1 and BP2 showed similar temperatures. As the controller shifted to HS2, the temperature at BP2 did not rise quickly, while BP1's temperature dropped slowly. This caused BP2's temperature to eventually exceed BP1's. According to our control design, temperature regulation was based on BP1, which was not intuitive. However, this choice avoided raising BP1 to ∼65°C when targeting 60°C at BP2, which could have damaged surrounding healthy tissue. This again highlights the importance of careful voxel selection for temperature control, as previously discussed. Additionally, the comparison of the clinical LITT scenario with proposed control system showed LC of ∼40% and ∼75% respectively. This showed that the proposed control system may increase the LC by ∼35%. However, the proposed control system did not achieve the target LC of 90%. It may be difficult to achieve targeted LC and current model is limited by the following factors:

- *Catheter cooling*

The maximum treatment temperature must be kept below 100°C to prevent tissue charring and vaporization,^9^ protect the laser catheter, and reduce the risk of system failure. To maintain this limit, the laser catheter is actively cooled during MRgLITT procedures. In this study, we applied a convective boundary condition at the tip of the laser probe to approximate this effect. However, accurately capturing the catheter's cooling performance requires a CHT modeling approach.

- *Heat sink effects from fluidic structures*

Fluidic structures, such as CSF and cerebral microvasculature near the tumor, act as heat sinks during MRgLITT. If the tumor is close to these structures, most of the supplied heat is dissipated through perivascular cooling^56^ effects. Accurately modeling these peri vascular cooling effects is crucial for predicting LC, as ignoring them could lead to an overprediction due to asymmetric tumor heating. In this study, heat sink effects were approximated by increasing the conductivity of fluid domains^34^ using the Nusselt number. However, the most accurate approach is CHT, which simultaneously solves fluid flow and heat transfer equations and computationally expensive. Studies indicate that CHT^57^, ^58^ has significant potential for predicting cooling effects in actual MRgLITT treatment.^56^

- *Temperature dependent thermal properties*

The model used in this study for temperature‐dependent thermal properties was capped at 90°C (Equation S9 and S10). While the model assumes an exponential increase in thermal properties beyond 90°C, experimental evidence shows that these properties actually decrease due to vaporization.^59^, ^60^ The effects of this decrease were not implemented in the current model, as their highly nonlinear behavior would substantially increase computational complexity. Moreover, in this study, temperatures above 90°C typically occur only near the laser tip, where tissue is already ablated once the boundaries have reached the desired damage threshold (controller case). Since less than 10% of the tumor volume experiences such temperatures for short durations (<1 min; Figures 6 and 7), this simplification is expected to have a minimal impact on overall model accuracy. Additionally, the controller likely compensates for these unmodeled effects, as demonstrated in Figures 6 and 7, where dynamic optical property variations were effectively mitigated with minimal influence on LC. In the tumor (negligible perfusion due to damage), the generated laser heat is primarily conducted outward towards the tumor boundary, further supporting the dominance of conductive heat transfer in maintaining accurate LC predictions which is a metric for treatment outcome.

- *Patient specific dynamic tissue properties*

These tissue properties are patient‐specific and may vary based on the nature of GBM^51^ (primary or metastatic). Newly diagnosed GBM may have significantly different properties compared to rGBM, as the latter has undergone radiation or other combination therapies. Radiation induces necrosis within the tumor core, altering the optical properties of the necrotic region, such as reduced light absorption. As a result, laser heat penetration decreases, limiting the heat source's reach to the tumor boundary.

Hence, it is essential to estimate both physical (thermal and optical) and physiological (blood perfusion) tissue properties to predict and control patient‐specific LC.^49^ An initial heating pulse, along with the cooling response, can serve as a surrogate to estimate^55^, ^61^, ^62^ these properties using inverse heat transfer. This pulse can also help verify the laser probe location during MRgLITT, enhancing safety, enabling adaptive treatment plan, and improving neuronavigation. Additionally, tissue properties change^51^, ^63^ during treatment due to thermal damage and tissue heterogeneity. Therefore, periodic evaluation of these properties is necessary to adapt the treatment plan and achieve the desired LC despite these uncertainties.

- *Heterogenous tumor geometry*

Tumor geometries are often heterogeneous, and when combined with the similar thermal and optical properties of tumor and surrounding tissues as well as the relatively small size of the full‐fire laser tip compared to the tumor, these factors lead to nearly spherical isothermal contours. This produces symmetric thermal damage centered on the laser tip, leaving irregular tumor regions undertreated. A potential solution is the use of side‐fire probes, which can better shape the ablation zone to match heterogeneous tumor geometries and thereby improve LC. While this approach may increase treatment time, the use of controlled heating strategies, as previously discussed, could help offset the added duration and allow the side‐fire laser to effectively treat regions that would otherwise remain undertreated.

- *Resolution and acquisition rate of MRTi*

In MRgLITT, MRTi typically uses 1 mm voxels. Because temperature is averaged within each voxel, there is effectively an unresolved rim of ∼0.5 mm at the tumor boundary. For a tumor of 2 cm diameter, that 0.5 mm rim corresponds to about 15% of the tumor volume that is not reliably monitored or controlled. As a result, the range of tumors that can be treated safely is effectively reduced, which can reduce LC. Improving MRTi resolution would shrink this boundary uncertainty and could improve LC.

Currently, clinical MRTi has an acquisition rate that introduces a time lag of approximately 5–7 s, which can affect controller performance. This delay makes it impractical to implement the proposed automated MRgLITT system in clinical settings. Recent studies have explored volumetric MRTi systems for MRgLITT, achieving an acquisition rate of 1 s^55^ with validation in preclinical settings. However, this system is not yet translated for clinical use.

Additionally, user select three MR imaging planes^9^ along the active laser probe (tip) to monitor temperature and thermal damage. As previously discussed, the maximum treatment temperature during MRgLITT is limited to 100°C to prevent vaporization. Some of the reported data from the studies report aggressive application of laser energies leading to localized temperatures greater than equal to 100°C.^9^ Exceeding this threshold can cause:

- Charring, reducing laser penetration depth in the lesion.
- Tumor shrinkage altering identified tumor margins.
- Generating air bubbles distorting MRTi images.

In summary, this study demonstrates the potential of an automated MRgLITT approach for incremental laser retraction in achieving targeted LC. Additionally, the side fire laser probe can also be utilized considering rotation for further treating asymmetric lesions. However, the designed control approach still lacks the ability to adapt to physiological and physical variations during treatment. This challenge can be addressed using predictive controllers, which rely on model‐based future predictions as well as adapting to real‐time feedback, reducing the risk of failure.

For clinical translation, extensive computational and experimental studies are required in future to develop patient‐specific treatment plan integrated with control system. Moving forward the focus should be on studying variable patient specific tissue properties based on de‐identified datasets, generating physics‐informed data for treatment planning. These models can be validated through phantom studies. Further preclinical validation, following the American Society of Mechanical Engineers Verification, Validation and Uncertainty Quantification framework,^64^ can be conducted using anthropomorphic human head phantoms with adjustable tissue properties and animal models to reduce the learning curve.

## 5 CONCLUSIONS

In this computational study we recommend use of light transport equation over beam heat source for modelling MRgLITT. The modelled control approach increased the LC by ∼35% with reduced treatment time. The automated control system has potential to reduce operator variability, improve end‐user experience and improve adaptation of MRgLITT for various applications. To ensure successful translation of the control system into clinic it is crucial to verify and validate the simulation modeled control approach.

## CONFLICT OF INTEREST STATEMENT

A.A., N.A., Y.S.L. and S.P. filed provisional patent applications for thermal dose‐control systems, and A. A. and Y. L., N. A. and S.P. have filed internal technology invention disclosures with Pennsylvania State University. C.H. is a consultant for Integra, NICO Corporation, Synaptive Medical, Stryker Corporation, True Digital Surgery, and Hemerion Therapeutics (unrelated to the project). R.I. is an inventor of several nanoparticle patents (unrelated to the project). All patents are assigned to either Johns Hopkins University or Aduro Biosciences, Inc. R.I. was a member of the Scientific Advisory Board of Imagion Biosystems. All other authors report no other conflicts of interest.

## Data Availability

The data that support the findings of this study are available on request from the corresponding author.
