# Supplementary material for: Automated laser retraction for targeted glioblastoma coverage during laser interstitial thermal therapy
Source: Med Phys. Author manuscript; Available in PMC 2026 Mar 6. (PMC12888946; doi:10.1002/mp.70267)
Supplement: Supplementry Information [file NIHMS2147510-supplement-Supplementry_Information.docx]

**Thermal dose feedback system with automated laser probe positioning for laser interstitial thermal therapy**

*Authors: Shreeniket Pawar^1,^* ***^‡^****; Yash Sharad Lad^,^****^‡,*^****; Nageshwar Arepally^1^; Ma’Moun Abu-Ayyad^1^; Robert Ivkov^2-5^, Brad E. Zacharia^6^; Constantinos Hadjipanayis^7^; Anilchandra Attaluri^1,*^*

**Affiliations:**

*^1^ Department of Mechanical Engineering, School of Science, Engineering, and Technology, The Pennsylvania State University—Harrisburg, Middletown, PA, USA*

*^2^* *Department of Radiation Oncology and Molecular Radiation Sciences, Johns Hopkins University School of Medicine, Baltimore, Maryland 21231, USA*

*^3^ Department of Oncology, Sydney Kimmel Comprehensive Cancer Center, Johns Hopkins University School of Medicine, Baltimore, MD, USA*

*^4^ Department of Mechanical Engineering, Whiting School of Engineering, Johns Hopkins University, Baltimore, MD, USA*

*^5^ Department of Materials Science and Engineering, Whiting School of Engineering, Johns Hopkins University, Baltimore, MD, USA*

^6^ *Department of Neurosurgery, Pennsylvania State Health, Hershey, PA 17033, USA.*

*^7^ Department of Neurological Surgery, University of Pittsburgh School of Medicine, Pittsburgh, PA 15213, USA*

*^‡^Equal contributions: Joint first authors*

*^*^ Correspondence:* [*aua473@psu.edu*](mailto:aua473@psu.edu) *(A.A.); sqp6127@psu.edu (S.P.)*

**Supplementary Information**

Modeling laser heat source

*i. Point source laser heating*

One approach we used to simulate the thermal response to the laser was based on the classical symmetric and isotropic solution to the light transport equation^1,2^. This method assumed a single point heat source located at the center of the laser tip and was referred to as the point heat source model. Although simplified, this model did not capture the actual spatial distribution of laser energy. The laser power was generated throughout the laser tip and not at a single point. Hence, this model provided an idealized representation of the heating effect. We used Equations (S1)-(S3) ^1,2^ to represent this case.

| $Q_{\mathrm{laser}}=P_{\mathrm{laser}}\cdot\mu_{\mathrm{eff}}^{2}\cdot\frac{exp(-\mu_{\mathrm{eff}}\cdot\left\Vert\vec{r}-\vec{r_{0}} \right\Vert)}{4\pi\cdot\left\Vert\vec{r}-\vec{r_{0}} \right\Vert}$ | (S1) |
| --- | --- |
| $\mu_{\mathrm{tr}}=\mu_{a}+\mu_{s}(1-g)$ | (S2) |
| $\mu_{\mathrm{eff}}=\sqrt{3\cdot\mu_{a}\cdot\mu_{\mathrm{tr}}}$ | (S3) |

where $P_{laser}$W is the laser input power, $\mu_{a}$ m^-1^, $\mu_{s}$ m^-1^ and g are the absorption probability, scattering probability of photons and anisotropic factor of the laser photon source respectively. $\left\| \vec{r}-\vec{r_{0}} \right\|$m denotes the distance from the heat source. This model reduces the parameter space to a single variable,$\mu_{eff}$, making it more computationally efficient while still capturing the essential trend of optical property changes with thermal damage.

*ii. Distributed Point Heat Sources in the laser tip*

Another approach we used to model the optical thermal response was based on the Huygens superposition principle**^3^**. In this method, each point along the laser tip was treated as a separate point heat source emitting isotropic irradiance as shown in Equation (S4) . Instead of assuming a single origin of heat, we distributed multiple heat sources along the tip to more accurately represent the actual energy deposition.

| $dE=P_{\mathrm{laser}}^{*}\cdot\frac{exp(-\mu_{\mathrm{eff}}\cdot\left\Vert\vec{r}-\vec{r_{0}} \right\Vert)}{4\pi\cdot\left\Vert\vec{r}-\vec{r_{0}} \right\Vert^{2}}\cdot d\vec{r}$ | (S4) |
| --- | --- |

where $dE$ W/m² is irradiance of isotropic differential point source, $P_{laser}^{*}$ W is the power of infinitesimally small heat source in the tip of the laser.

The total heat source was applied by integrating the contribution from each point source over the tip volume as shown in Equation (S5)**^3^**. To implement this computationally, we approximate the continuous distribution using a finite number of discrete sources. Specifically, we modeled 15 equally spaced points along the axis of the laser tip. Each of these points acted as the center of a point heat source as shown in Equation (S6). Due to the symmetry of the laser, we neglected radial variations and considered only axial distribution. This approach provided a more realistic representation of the volumetric heat deposition from the laser, capturing the distributed nature of the energy release along the tip.

| $Q_{\mathrm{laser}}=\iiint_{\mathrm{Tip}} \frac{P_{\mathrm{laser}}\cdot\mu_{\mathrm{eff}}^{2}}{V_{\mathrm{tip}}}\cdot\frac{exp(-\mu_{\mathrm{eff}}\cdot\left\Vert\vec{r}-\vec{r_{0}} \right\Vert)}{4\pi\cdot\left\Vert\vec{r}-\vec{r_{0}} \right\Vert^{2}}\cdot dV$ | (S5) |
| --- | --- |
| $Q_{\mathrm{laser}}=P_{\mathrm{laser}}\cdot\mu_{\mathrm{eff}}^{2}\cdot\frac{\exp\left( -\mu_{\mathrm{eff}}\cdot\left\Vert\vec{r}-\vec{r_{0,i}} \right\Vert\right)}{n\cdot4\pi\cdot\left\Vert\vec{r}-\vec{r_{0,i}} \right\Vert}i\epsilon1,2,\ldots,n$ | (S6) |

where $V_{tip}$ m³ is the volume of the tip, $dV$ m³ is the infinitesimally small volumetric element, which is integrated over the volume of the tumor, $i$ is the heat source on the axis of the laser tip and $n$ are the equidistant heat source on the tip of the laser.

Both approaches yield the laser power and power deposited is same when the heat source is integrated over an infinite domain, assuming a constant effective attenuation coefficient ($\mu_{eff}$). Since the heat source decays exponentially with distance, energy conservation holds within 1% of the laser power when the distance from the source reaches approximately $\left\| \vec{r}-\vec{r_{0}} \right\|=4/\mu_{eff}$. Previous studies have estimated $\mu_{eff}$ based on damage-dependent optical properties using experimental data^3^. In our study, we modeled damage-dependent optical properties (details provided later), but we did not have the experimental data. Under these conditions, the deposited power varied because the effective attenuation coefficient $\mu_{eff}$ increased near the probe tip, approaching the coagulated tissue value ${(\mu}_{eff,c})$, and remained approximately constant farther from the tip, matching the native tissue value ($\mu_{eff,n})$. This variation led to an overestimation of the deposited laser power. To address this and ensure energy conservation, we introduced a scaling factor (SF), as described in Equation (S7).

| $SF=\iiint_{\mathrm{dV}} \mu_{\mathrm{eff}}^{2}\cdot\frac{exp(-\mu_{\mathrm{eff}}\cdot\left\Vert\vec{r}-\vec{r_{0}} \right\Vert)}{4\pi\cdot\left\Vert\vec{r}-\vec{r_{0}} \right\Vert}\cdot r^{2}\cdot sin(\phi)\cdot dr\cdot d\theta\cdot d\phi$ | (S7) |
| --- | --- |

The modified heat source is shown in Equation (S8).

| $Q_{\mathrm{laser}}=\frac{P_{\mathrm{laser}}\cdot\mu_{\mathrm{eff}}^{2}}{\mathrm{SF}}\cdot\frac{exp(-\mu_{\mathrm{eff}}\cdot\left\Vert\vec{r}-\vec{r_{0}} \right\Vert)}{4\pi\cdot\left\Vert\vec{r}-\vec{r_{0}} \right\Vert}$ | (S8) |
| --- | --- |

We similarly modified distributed point heat source to keep deposited power the same. We compared the results between LPHS and DPHS and if the RMS value of temperature at CP1-CP3 and temperature and thermal dose at BP1-BP3 are within 5 % we will use the LPHS for further modelling.

Temperature dependent thermal properties

The temperature-dependent properties used for the brain and tumor are described by the exponential expressions in Equation (S9) and (S10)^4^. The thermal properties were seized at 90 °C as the model used is described till 90 °C and tissue thermal properties have been shown to have inverse relationship with temperature above 90 °C which is not captured in the current model^5^. Additionally, this approach decreases the computational complexity for the study.

| $k_{i}(x,y,z,t)=min\left( a_{k,i}+b_{k,i}\cdot\exp\left( c_{k,i}\cdot T\left( x,y,z,t \right) \right),a_{k,i}+b_{k,i}\cdot\exp\left( c_{k,i}\cdot T_{upper} \right) \right)$ | (S9) |
| --- | --- |
| $\mathrm{VHC}_{i}(x,y,z,t)=min\left( a_{VHC,i}+b_{VHC,i}\cdot\exp\left( c_{VHC,i}\cdot T\left( x,y,z,t \right) \right),a_{VHC,i}+b_{VHC,i}\cdot\exp\left( c_{VHC,i}\cdot T_{upper} \right) \right)$ | (S10) |

where $a_{k,i}$ W(m$\boldsymbol{\cdot}$K)^-1^, $b_{k,i}$ W(m$\boldsymbol{\cdot}$K)^-1^, $c_{k,i}$ K^-1^, $a_{VHC,i}$J(m³$\boldsymbol{\cdot}$K)^-1^, $b_{VHC,i}$J(m³$\boldsymbol{\cdot}$K)^-1^ and $c_{VHC,i}$ K^-1^ are the regression constants for the expression of $k_{i}(x,y,z,t) W{(m\boldsymbol{\cdot}K)}^{-1}$, ${VHC}_{i}\left( x,y,z,t \right) or a_{VHC,i}$ J(m³$\boldsymbol{\cdot}$K)^-1^ =$\rho_{i}C_{p,i}$is the volumetric heat capacity which was obtained as product of density and specific heat capacity for brain and tumor and $T_{upper}$ was constrained at 363.15 [K]. In this study we used the $a_{k,i}$ and $a_{VHC,i}$ as the constant value we used in our previous study^6^ and assuming that the change in thermal properties would be similar to brain of the calf we used $b_{k,i}$ $c_{k,i}$, $b_{VHC,i}$ and $c_{VHC,i}$ from study in calf^4^. The parameters used are shown in Table S1 and Table S2.

Table S1: Constant thermal properties for different domains of the human head anatomy.

| Part | Heat capacity, $C_{p}$  $J\left( \mathrm{kg}\cdot K \right)^{-1}$ | Density, $\rho$ $\mathrm{kg}\cdot m^{-3}$ | Thermal Conductivity, $k$  $W\left( m\cdot K \right)^{-1}$ | Ref. |
| --- | --- | --- | --- | --- |
| Skull | 1313 | 1908 | 0.32 | 7-9 |
| CSF (general and ventricles) | 4096 | 1007 | 0.62 | 7,10 |
| Blood | 3617 | 1050 | 0.52 | 7-9 |
| Laser probe (Silica) | 750 | 2200 | 1.38 | 11 |

Table S2: Temperature dependent thermal properties for brain and tumor used in the study.

| **Part** | **Tumor** | **Brain** | **Ref.** |
| --- | --- | --- | --- |
| $a_{k,i}$ W(m$\boldsymbol{\cdot}$K)^-1^ | 5.70×10^-1^ | 5.10×10^-1^ | 9 |
| $b_{k,i}$ W(m$\boldsymbol{\cdot}$K)^-1^ | 2.26×10^-9^ | 2.26×10^-9^ | 4 |
| $c_{k,i}$ K^-1^ | 2.08×10^-1^ | 2.08×10^-1^ | 4 |
| $a_{VHC,i}$J(m³$\boldsymbol{\cdot}$K)^-1^ | 3.91×10^6^ | 3.80×10^6^ | 7,8 |
| $b_{VHC,i}$J(m³$\boldsymbol{\cdot}$K)^-1^ | 9.53×10^-6^ | 9.53×10^-6^ | 4 |
| $c_{VHC,i}$ K^-1^ | 2.40×10^-1^ | 2.40×10^-1^ | 4 |

Thermal damage dependent optical properties

*i. Multi-optical parameter*

This method models the absorption coefficient $\mu_{a}$ m^-1^, scattering coefficient $\mu_{s}$ m^-1^ and anisotropy factor *g* separately as shown in Equations (S11)-(S13)^12^.

| $\mu_{a}= \mu_{a,n}\cdot\left( 1-\Omega\right)+\mu_{a,c}\cdot\Omega$ | (S11) |
| --- | --- |
| $\mu_{s}= \mu_{s,n}\cdot\left( 1-\Omega\right)+\mu_{s,c}\cdot\Omega$ | (S12) |
| $g= g_{n}\cdot\left( 1-\Omega\right)+g_{c}\cdot\Omega$ | (S13) |

where $\mu_{a,n}$ m^-1^, $\mu_{s,n}$ m^-1^ and $g_{n}$ are native tissue properties while $\mu_{a,c}$ m^-1^, $\mu_{s,c}$ m^-1^, and $g_{c}$ represent coagulated tissue properties. The term $\Omega(x,y,z,t)$ denotes spatial temporal varying thermal damage. The native tumor properties were obtained from the measured properties as shown in Table S3. To model the coagulated tumor properties we assumed that absorption and scattering co-efficient are 50 % higher than native properties and coagulated anisotropic factor was 0.01 less than native anisotropic factor. These assumptions were based on optical properties changes in coagulated and native tissue in other parts of the brain^13^.

*ii. Single optical parameter*

To reduce complexity, optical properties can be combined into a single effective absorption coefficient as shown in Equations (S14)-(S18)^14^. This approach simplifies the model at the cost of accuracy.

| $\mu_{tr,n}=\mu_{a,n}+\mu_{s,n}(1-g_{n})$ | (S14) |
| --- | --- |
| $\mu_{eff,n}=\sqrt{3\cdot\mu_{a,n}\cdot\mu_{tr,n}}$ | (S15) |
| $\mu_{tr,c}=\mu_{a,c}+\mu_{s,c}(1-g_{c})$ | (S16) |
| $\mu_{eff,c}=\sqrt{3\cdot\mu_{a,c}\cdot\mu_{tr,c}}$ | (S17) |
| $\mu_{\mathrm{eff}}= \mu_{eff,n}\cdot\left( 1-\Omega\right)+\mu_{eff,c}\cdot\Omega$ | (S18) |

Table S3: Optical parameters for the laser irradiated tissue.

| **Property** | **Value** | | | **Ref** |
| --- | --- | --- | --- | --- |
|  | **Lower** | **Nominal** | **Upper** |  |
| $\mu_{a,n}$mm^-1^ | 1.88×10^-2^ | 5.13×10^-2^ | 8.75×10^-2^ | 13 |
| $\mu_{s,n}$mm^-1^ | 4.34 | 7.41 | 1.11×10^1^ | 13 |
| $g_{n}$ | 9.58×10^-1^ | 9.62×10^-1^ | 9.66×10^-1^ | 13 |
| $\mu_{eff,n}$mm^-1^ | 9.69×10^-2^ | 2.26×10^-1^ | 3.81×10^-1^ | 13 |
| $\mu_{a,c}$mm^-1^ | 2.82×10^-2^ | 7.69×10^-2^ | 1.31×10^-1^ | - |
| $\mu_{s,c}$mm^-1^ | 6.51 | 1.12×10^1^ | 1.67×10^1^ | - |
| $g_{c}$ | 9.48×10^-1^ | 9.52×10^-1^ | 9.56×10^-1^ | - |
| $\mu_{eff,c}$mm^-1^ | 1.63×10^-2^ | 3.75×10^-1^ | 6.27×10^-1^ | - |

**COMSOL Multiphysics – MATLAB Simulink Simulation Parameters:**

Table S4: Bioheat transfer properties for COMSOL Multiphysics Simulation.

| **Description** | **Value** |
| --- | --- |
| Convective Heat Transfer Coefficient Skull | 10 [W/(m^2^·K)] |
| Nusselt's Number CSF | 1.725 |
| Metabolic Heat Rate Brain | 10373.5 [W/m^3^] |
| Metabolic Heat Rate CSF | 0 [W/m^3^] |
| Maximum Laser Power | 15 [W] |
| Metabolic Heat Rate Skull | 70 [W/m^3^] |
| Metabolic Heat Rate Tumor | 25000 [W/m^3^] |
| Universal Gas Constant | 8.314 [J/mol/K] |
| Blood Temperature | 310.15 [K] |
| Ambient Temperature | 293.15 [K] |
| Perfusion rate Brain | 0.0085 [1/s] |
| Perfusion rate Skull | 0.000143[1/s] |
| Perfusion rate Tumor | 1.72×10^-3^[1/s] |
| Activation energy Tumor | 2.38×10^5^ [J/mol] |
| Frequency factor Tumor | 1.8×10^36^ |

**COMSOL Multiphysics – MATLAB Simulink Simulation Workflow:**

1. Geometry:

- Loaded mesh files of the deidentified patient dataset into COMSOL Multiphysics.
- Six domains were formed using the mesh files viz., skull, CSF general, brain, CSF ventricles (2), tumor and laser.
- Laser was modeled using cylindrical geometry within the tumor in COMSOL Multiphysics.

1. Materials:

- Appropriate material properties were assigned to different domains, as given in Table S1 and Table S2.

1. Bioheat Transfer Modeling:

- Used the Bioheat Transfer module to simulate Pennes’ bioheat equation.
- Modeled laser (ω=0 [s^-1^]) as solid biological tissues.
- Modeling CSF:

The heat sink effect of the CSF was modeled using convectively enhanced conductivity^15-18^, where the Nusselt number was used to represent the characteristics of the CSF. This modeling approach provides heat sink effects that are approximate to those of the conjugate heat transfer (CHT) analysis, with a significantly lower computational burden. Equation (S19) was used to compute the Prandtl number (*Pr*) where *C_p_* is the specific heat capacity [J(kg$\boldsymbol{\cdot}$K)^-1^], $\mu$ is the dynamic viscosity [Pa$\boldsymbol{\cdot}$s] and *k* is the thermal conductivity [W(m$\boldsymbol{\cdot}$K)^-1^] of the CSF. The Grashof number (*Gr*) was calculated using Equation (S21), where *Ra* is the Rayleigh number. These dimensionless numbers were used to calculate the Nusselt number, *Nu*, using Equation (S22), with the assumption that the angle of inclination of the CSF ventricle from the vertical (*θ*) is 30º.

| $\Pr= \frac{\mu\boldsymbol{\cdot}C_{p}}{k}$ | (S19) |
| --- | --- |
| $\mathrm{Ra} = \frac{L^{3}\boldsymbol{\cdot}\rho^{2}\boldsymbol{\cdot}g\boldsymbol{\cdot}\beta\boldsymbol{\cdot}\Delta T\boldsymbol{\cdot}C_{p}}{\mu\boldsymbol{\cdot}k}$ | (S20) |
| $Gr=\frac{Ra}{Pr}$ | (S21) |
| $Nu={0.67[Gr\boldsymbol{\cdot}\Pr\boldsymbol{\cdot}\cos\left( \theta\right)]}^{0.25}$ | (S22) |
| $q= -k\boldsymbol{\cdot}\mathrm{Nu}\boldsymbol{\cdot}\nabla T$ | (S23) |

Equation (S23) gives the convective heat transfer between the CSF and the tumor using the Nusselt number. The properties of the fluid, along with the numerical values for the *Pr*, *Ra*, *Gr*, and *Nu*, are given in Table S5.

Table S5: Cerebrospinal Fluid properties and calculation of Nusselt Number.

| **Property** | **Value** |
| --- | --- |
| CSF velocity, $u$ | $0.08 [m\cdot s^{-1}]$ |
| CSF thermal conductivity, $k$ | $0.62 [W\left( m\cdot K \right)^{-1}]$ |
| CSF specific heat capacity, $C_{p}$ | $4096 [ J\left( \mathrm{kg}\cdot K \right)^{-1}]$ |
| CSF dynamic viscosity, $\mu$ | $7.84\times{10}^{-4} [\mathrm{Pa}\cdot s]$ |
| CSF Prandtl number, $P_{r}$ | $5.179$ |
| CSF Rayleigh number, $R_{a}$ | $4.79\times{10}^{5}$ |
| CSF Grashof number, $G_{r}$ | $9.27\times{10}^{4}$ |
| CSF Nusselt number, $N_{u}$ | $1.725$ |

- Modeling thermal damage and microvascular perfusion:

In this study, MRgLITT treatment was assumed to be carried out using the Medtronic Visualase™ system, which uses the Arrhenius model to calculate thermal damage^19^. For the simulations, we modeled the thermal damage using the Arrhenius model, as represented by Equation (S24). Here, *DS* is the degree of microvascular damage over time, *A* is the frequency factor [s^-1^], *E_a_*is is the activation energy [J$\boldsymbol{\cdot}$mol^-1^], *R* is the universal gas constant [J(K$\boldsymbol{\cdot}$mol)^-1^], *and T(t)* is the absolute tissue temperature [K] as a function of time. The degree of vascular damage varies between 0 (no damage) and 1 (complete damage).

| $DS=1-\exp\left( -A\int_{0}^{t} e^{-\frac{E_{a}}{\mathrm{RT}\left( t \right)}} \right)\mathrm{dt}$ | (S24) |
| --- | --- |

Hence, thermal damage dependent blood perfusion^20-25^ modeled within the tumor can be represented by $\omega_{b,tumor}$ in Equation (S25).

| $\omega_{b,tumor}\left( T \right)=\left\{ \begin{aligned} \omega_{b,tumor}\left( 30DS+1 \right), \left( DS\leq0.02 \right) \\ \omega_{b,tumor}\left( -13DS+1.86 \right), \left( 0.02<DS\leq0.08 \right) \\ \omega_{b,tumor}\left( -0.79DS+0.884 \right), \left( 0.08<DS\leq0.97 \right) \\ \omega_{b,tumor}\left( -3.87DS+3.87 \right), \left( 0.97<DS\leq1.0 \right) \end{aligned} \right.$ | (S25) |
| --- | --- |

The values of perfusion rates for different domains and values of *A* and *E_a_* used in the present study are in Table S6.

| Table S6: Arrhenius parameter values and blood perfusion rates for different domains.   \| **Property** \| **Value** \| \| --- \| --- \| \| Blood Temperature, *T_b_* \| $310.15 [K]$ \| \| Blood Perfusion in Skull, $\omega_{b,skull}$ \| $70 [s^{-1}]$ \| \| Blood Perfusion in Brain, $\omega_{b,brain}$ \| $8.5\times{10}^{-2} [s^{-1}]$ \| \| Blood Perfusion in Tumor, $\omega_{b,tumor}$ \| $1.72\times{10}^{-3} [s^{-1}]$ \| \| Activation Energy, *E_a_* \| $2.38\times{10}^{5}$ $[J\cdot\mathrm{mol}^{-1}]$ \| \| Frequency Factor, *A* \| $1.8\times{10}^{36}$ $[s^{-1}]$ \| |
| --- | --- | --- | --- | --- | --- | --- | --- | --- | --- | --- | --- | --- | --- | --- |

1. Laser Heating:

- Set up three distinct surface heat sources within the bioheat transfer module for three different laser heat sources with diameter as 1.65 [mm] and length as 5 [mm].

1. Thermal probes and measurements:

- Modeled blood perfusion using domain ordinary differential equations (ODEs) module.
- Strategically configured temperature and thermal damage measuring probes based on open loop simulations to maximize thermal dose coverage and minimize healthy tissue damage:
  - Boundary point probes (BP1, BP2 and BP3) placed at three specified points on the tumor boundary.
  - Domain probe measuring maximum laser temperature was positioned within the cylindrical laser source.

1. Mesh Convergence:

- Mesh convergence was carried out using physics-controlled mesh with three different mesh sizes normal, fine and finer.
- Based on the results of mesh convergence, physics-controlled mesh was selected in COMSOL Multiphysics with fine mesh size.

1. Solver Settings:

- Times to store: Output times by interpolation
- Relative tolerance: 0.01
- Absolute tolerance:
  - Global method: Scaled
  - Tolerance method: Factor
  - Tolerance factor: 0.1
  - Update scaled absolute tolerance: On
- Time-Dependent Solver:
  - Solver type: Implicit
  - Method: Backward Differentiation Formula (BDF)
  - Steps taken by solver: Strict
  - Maximum step constraint: Automatic
  - Maximum BDF order: 2
  - Minimum BDF order: 1
  - Event tolerance: 0.01
  - Consistent initialization: Backward Euler
  - Fraction of initial step for Backard Euler: 0.001
  - Safety factor for Backward Euler: 20
- Direct Solver:
  - Solver typer: Multifrontal Massively Parallel Solver (MUMPS)
  - Memory allocation factor: 1.2
- Direct, Heat transfer Solver:
  - Solver type: PARallel DIrect SOlver (PARDISO)

1. Modelling laser log from de-identified log as shown in Figure 2.
   - Beam heat source:
     1. Divided laser into three distinct cylinders.
     2. Fuzzy logic was used to control the position of the source using conditional if else.
     3. Piecewise function was used to model the temporal on and off the laser.
   - Volumetric heat source:
     1. Divided tumor into five parts with middle three of 5 [mm] each.
     2. Fuzzy logic was used to control the position of the source using conditional if else.
2. Proportional-Integral-Derivative (PID) Control:

- Employed COMSOL Livelink for Simulink within COMSOL Multiphysics and MATLAB Simulink to conduct PID-controlled heating simulations^26^.
- Configured the beam heat source as power an input for the Simulink co-simulation block, with temperature and Arrhenius thermal damage probes (CP, P1, P2, P3) serving as outputs.
- Routed the co-simulation block’s output to the PID block input, including a switch to manage and limit the maximum temperature at the tumor boundary probes (P1, P2, P3) to 55°C and to control the tumor center temperature (CP) below 110°C.
- Defined controller safety criterion using a switch such that, if any of the tumor boundary probe (P1, P2, P3) reached a thermal damage of 0.99, the input to the co-simulation block would automatically turn OFF.
- The output of the PID block was applied to the input of co-simulation block with the safety criterion.
- Three different study steps, similar to the pulsed heating method were utilized to simulate the spatially confined MNP heating.

**Mesh Convergence**

To verify that the model was independent of mesh variation, a mesh convergence study was conducted for three different mesh sizes in the FEA software: normal, fine, and finer, with assuming constant optical properties. A physics-controlled mesh was utilized, which automatically adjusted to high mesh density for the regions of interest and relaxed mesh density for the remaining regions. The mesh convergence criteria were established based on the acceptable temperature variation of ±0.01 [°C], aligning with the precision of the temperature sensor.

Table S7 lists the computational statistics for different mesh sizes. There was little variation in temperature with mesh size. However, to account for the nonlinear thermal dose, an optimum between the mesh size and simulation time was chosen. Therefore, a fine mesh was used for further simulations as the temperature variation was within ±0.2 [°C].

Table S7: Mesh convergence results for different mesh sizes.

| **Parameters** | **Normal Mesh** | **Fine Mesh** | **Finer Mesh** |
| --- | --- | --- | --- |
| Number of mesh elements | 48478 | 96602 | 232696 |
| Treatment time [s] | 100 | 100 | 100 |
| Computational time [s] | 820 | 1365 | 1915 |
| Calculated temperature at CP1 [°C] | 43.58 | 43.58 | 43.57 |

**Verification**

Methods

The COMSOL solver was verified using a line heat source, based on the Pennes’ bioheat equation with zero perfusion. The geometry and boundary conditions employed in the study are illustrated in Figure S1. An axisymmetric cylindrical geometry was utilized, with a cylinder length of 1.5 cm and a radius of 1 [m]. A boundary condition was applied at a radius of 1 [m], assuming negligible temperature change, as this radius exceeds the heat sources radius of influence. Adiabatic conditions were imposed at the cylinder ends (0 [cm] and 1.5 [cm]). Symmetry was enforced at the central axis (radius = 0 [m]). A line heat source of 10 [W/cm] was introduced along the center of the domain, with an initial temperature of 37 [°C] across the domain.


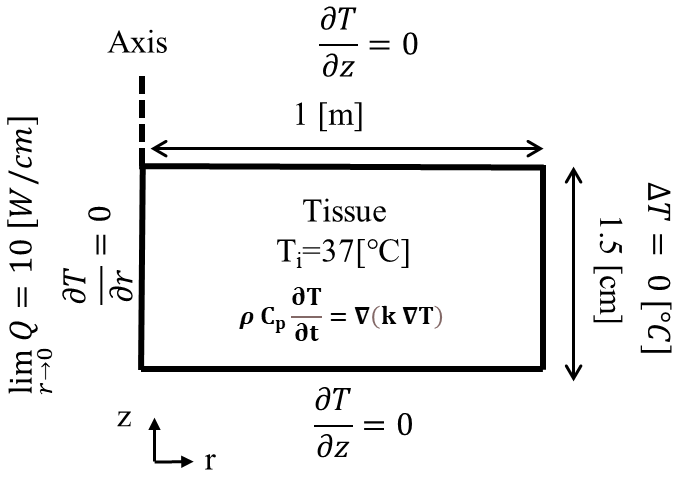


Figure S1: Geometry and boundary conditions for the verification of the numerical results (Not to scale).

The analytical solution^27^ for the problem is shown in equation (S26).

| $\Delta T(r,t)=\frac{\dot{Q}}{4\pi kL}\mathrm{Ei}\left( \frac{\rho\cdot C_{p}\cdot r^{2}}{4\cdot k\cdot t} \right)$ | (S26) |
| --- | --- |

where $\Delta T(r,t)$ [°C] is the change in temperature, $\dot{Q}$ [W/m] is the heat source per unit length, $k$ [W/(m·°C)], $L$ [m] is the length of the cylinder, $\mathrm{Ei}$ is the exponential integral, $\rho$ [kg/m^3^] is the density, $C_{p}$[J/(kg·°C)] is the specific heat, $r$ [m] is the distance from the center and $t$[s] is the time.

| $Ei(x)=\int_{-\infty}^{x} \frac{e^{t}}{t}\mathrm{dt}$ | (S27) |
| --- | --- |

The numerical solution for the problem was solved using the numerical scheme, time step, mesh size and solver settings. The material properties for the verification were density of 1056 [kg/m^3^], specific heat of 3700 [J/(kg·°C)] and conductivity of 0.57 [W/(m·°C)].

**Validation**

Methods

Validation was performed on: (1) previous validation study for LITT by Fuentes et al. and (2) a pre-clinical study of PID controller for LITT^28,29^. For both cases, we modelled the Pennes bioheat equation as shown in Equation 1,2 and constant optical properties. The simplified geometry for the validation is shown in Figure S2 and material properties used for the study are shown in Table S8. For case 1, we used two perfusions viz. lower (1.397×10^-2^ s^-1^) and higher (2.794×10^-2^ s^-1^) perfusion to account for the heterogenous perfusion considered in the perfusion model. For case 2, we used diffusivity and absorption co-efficient of 0.18 mm^2^s^-1^ and 0.65 °C·(s·W)^-1^ as obtained by authors [29].


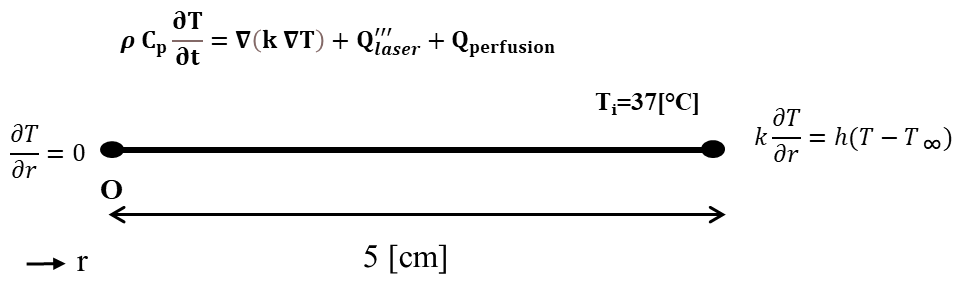


Figure S2: Simplified geometry for the validation of the FEA model.

Table S8: Material properties for the validation study.

| Study | Part | Heat capacity, $C_{p}$  $J\left( \mathrm{kg}\cdot K \right)^{-1}$ | Density, $\rho$ $\mathrm{kg}\cdot m^{-3}$ | Thermal Conductivity, $k$  $W\left( m\cdot K \right)^{-1}$ | Ref. |
| --- | --- | --- | --- | --- | --- |
| Canine Prostate | Prostate | 3600 | 1045 | 0.53 | 7-9,28 |
|  | Blood | 3840 | 1050 | - | 7-9,28 |
| Pig Muscle | Muscle | 3421 | 1090 | 0.8576 | 7-9,29 |

**Results**

Verification

The change is temperature is compared at 4 radii viz. 1 ,1.2, 1.4 and 1.6 [cm] respectively at length of 0.75 [cm] for numerical and analytical solution. The results show that the numerical solution agrees with the analytical solution. The radii were chosen as these are approximately the distance of the boundary of the tumor from the heat source.


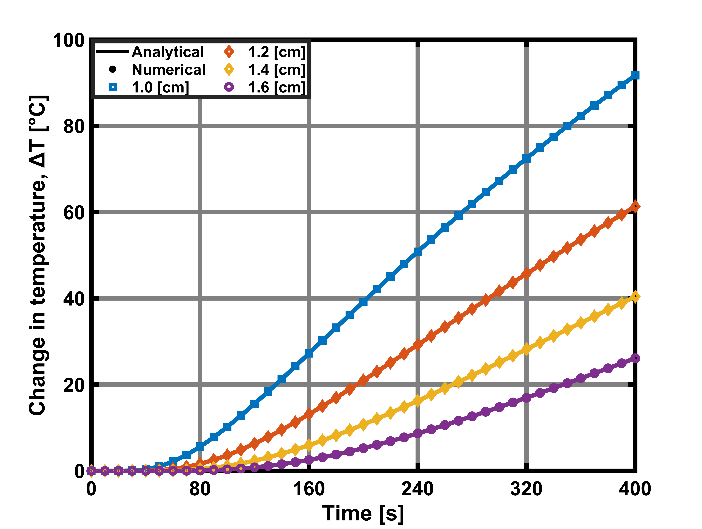


Figure S3: Comparison between the analytical and numerical solutions.

Validation of FEA model

Case 1

The model validation is shown in Figure S4 at three planes corresponding to those in Figure 7 of Fuentes et al., at 55 [s] of laser on-time [28]. The magnetic resonance temperature imaging (MRTi) data and finite element analysis (FEA) results from Fuentes et al. are bounded within our simulation results across all three planes. While Fuentes et al. incorporated heterogeneous thermal properties in their FEA model, our approach assumes constant properties, which may contribute to discrepancies. Additionally, our assumptions regarding thermal conductivity and perfusion further influence the differences in the results.


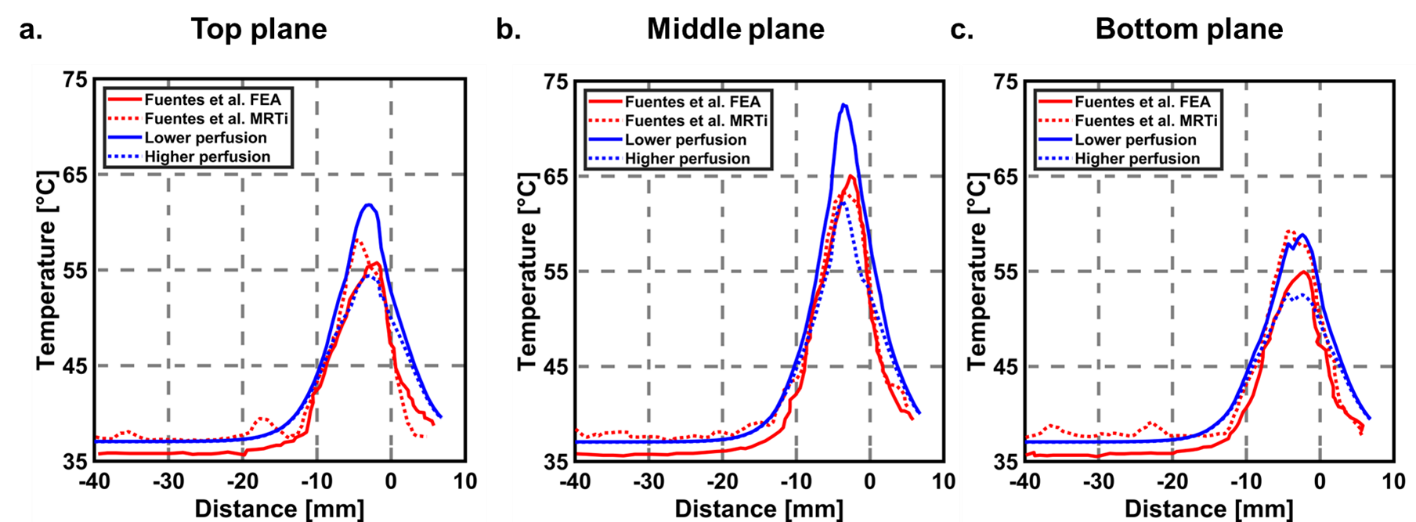


Figure S4: Comparison of the results between Fuentes et al. and our model at three planes at 55 [s] [28]. a. Top plane; b. Middle plane and c. Bottom plane.

Case 2:

We validated our model for the pig muscle study by Desclides et al. (Figure 4 and 5 from their work) [29]. We first compared our model against a constant power of 2 W for 30 s for a voxel of maximum temperature as shown in Figure S5 a. Maximum temperature deviation of ~1.5 °C was observed with the given parameters. Figure S5 b and c temperature control using PID controller with noise obtained by Desclides et al. The temperature obtained by our model showed agreement with Desclides et al.


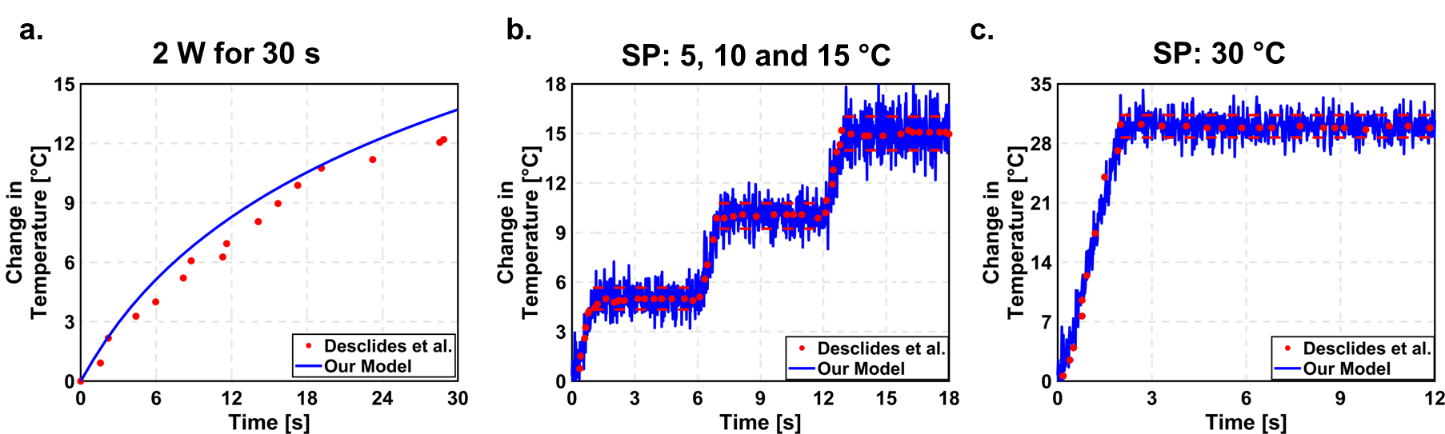


Figure S5: Comparison of the results between Desclides et al. and our model for open loop and controlled temperature [29]. a. 2 W for 30 s; b. Setpoint of 5, 10 and 15 °C for 5 min each and c. Setpoint of 30 [°C] for 10 min.

Comparison of optical parameter


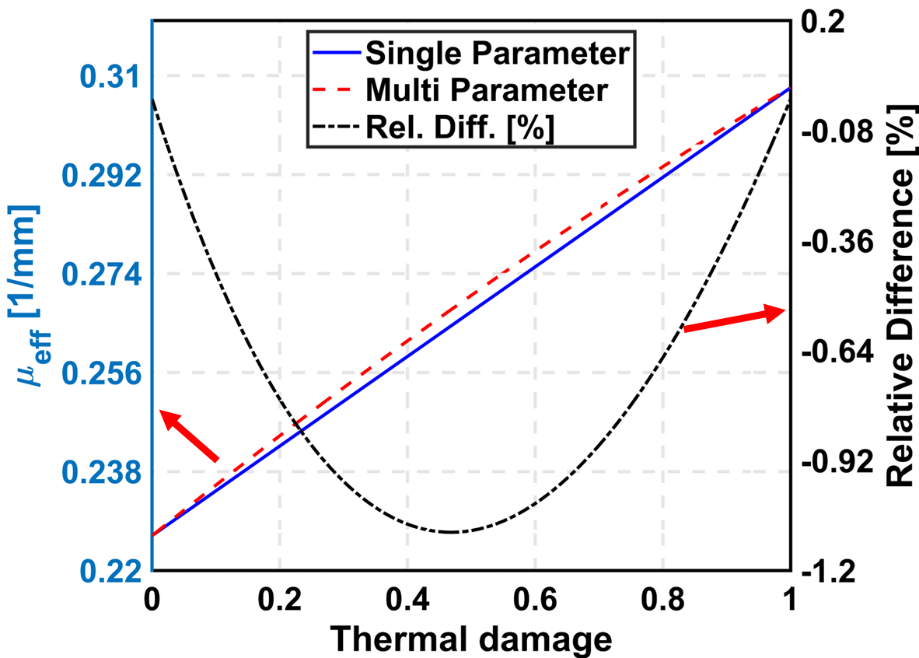


Figure S6: Attenuation co-efficient and relative difference between multiple and single parameter modelling as a function of thermal damage.

Comparison between laser heat source modelling


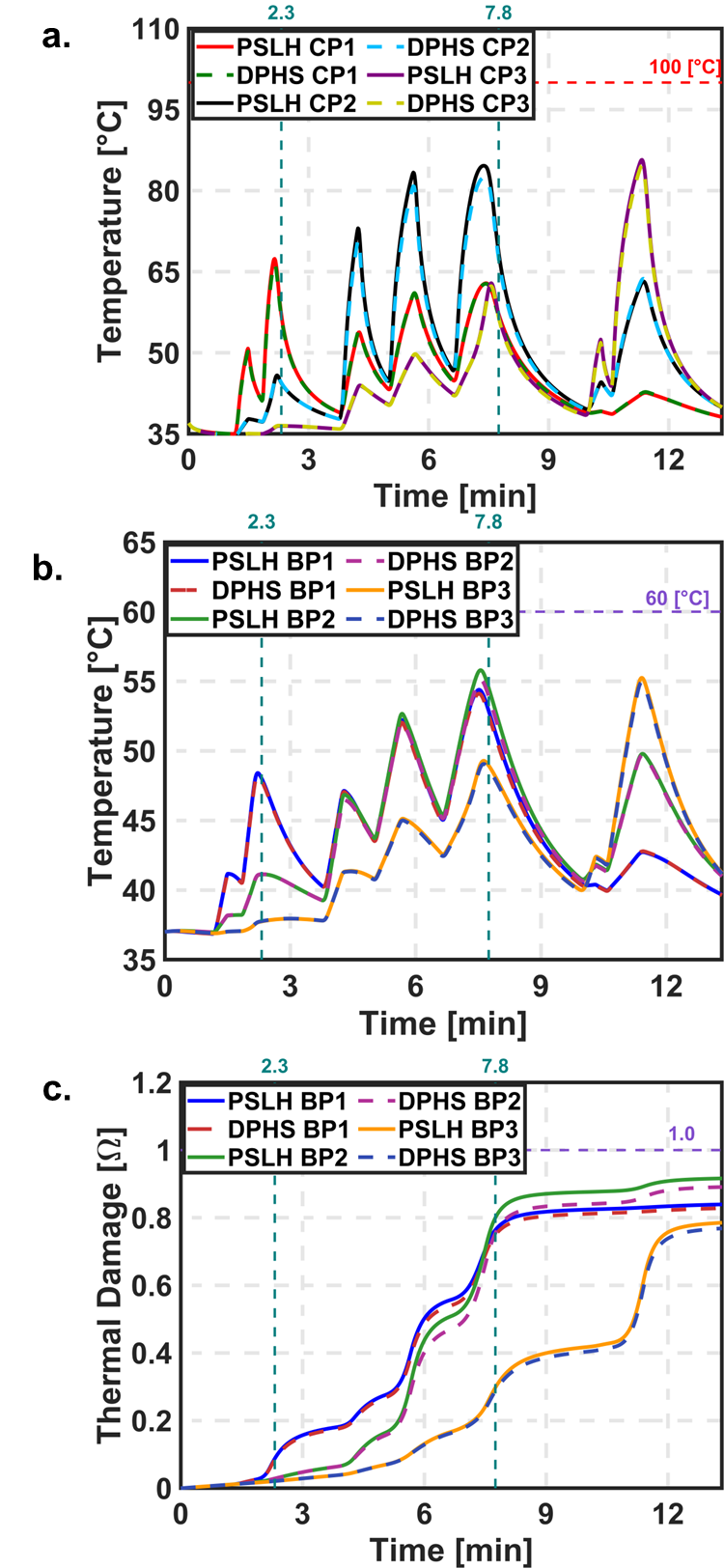


Figure S7: Comparison between temperature and thermal damage at CP and BP for point source laser heating (PSLH) and distributed point heat source (DPHS). a. Temperature at CP. b. Temperature at BP. c. Thermal damage at BP.

Table S9: Performance metrics for comparison between PSLH and DPSH methods.

| **Parameters** | | **BP1** | **BP2** | **BP3** |
| --- | --- | --- | --- | --- |
| Root mean square error | Temperature at CP [°C] | 0.2173 | 0.8964 | 0.3299 |
|  | Temperature at BP [°C] | 0.1286 | 0.2709 | 0.1252 |
|  | Thermal damage at BP | 0.0107 | 0.0271 | 0.0101 |
| Maximum Error | Temperature at CP [°C] | 0.9870 | 2.5490 | 1.2210 |
|  | Temperature at BP [°C] | 0.2950 | 0.7080 | 0.3020 |
|  | Thermal damage at BP | 0.0168 | 0.0475 | 0.0199 |

Parametric sweep on attenuation co-efficient for constant optical properties


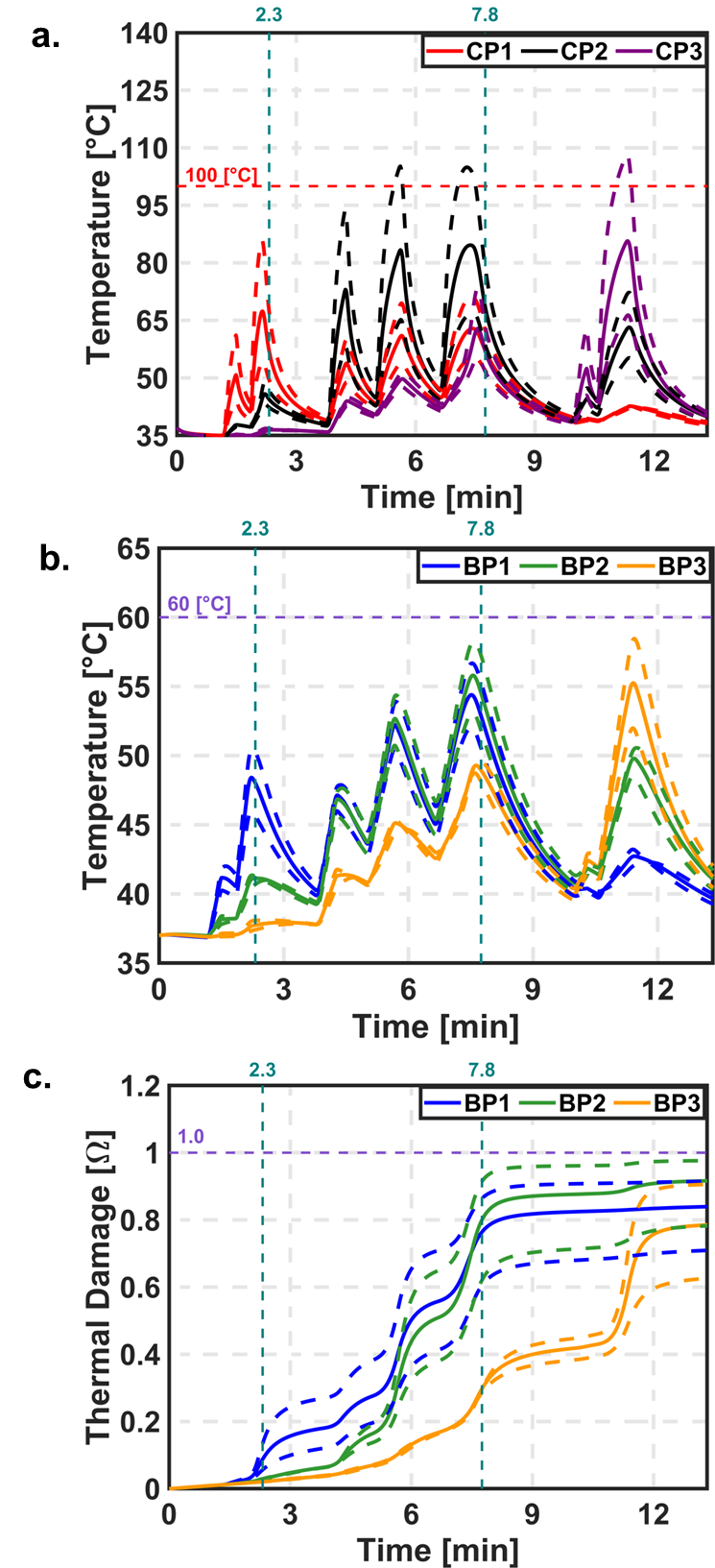


Figure S8: Parametric sweep on attenuation co-efficient for constant optical properties. Comparison between temperature and thermal damage at CP and BP. a. Temperature at CP. b. Temperature at BP. c. Thermal damage at BP.

Uncertainty Propagation

*Input Uncertainty parameter*

Table S10: Input parameters for the uncertainty quantification using SOBOL method for two independent parameters of attenuation co-efficient.

| Parameter | Distribution | Mean m^-1^ | Standard deviation m^-1^ |
| --- | --- | --- | --- |
| Native attenuation co-efficient | Normal | 234.82 | 142.38 |
| Coagulated attenuation co-efficient | Normal | 388.37 | 231.98 |


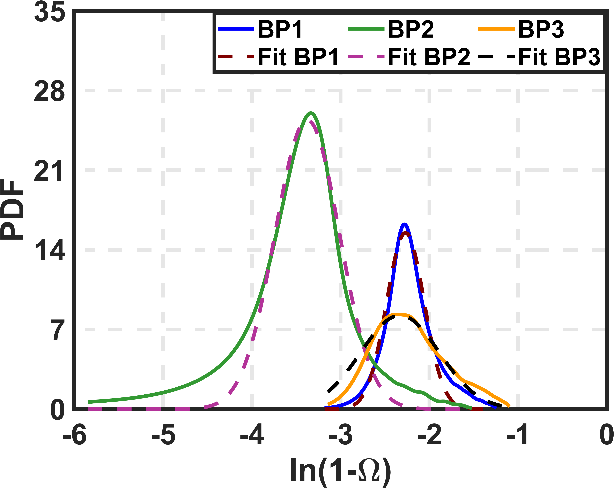


Figure S9: Logarithm of probability of survival fitted with normal distribution shows agreement showing the potential to use of logarithmic model as surrogate model for uncertainty propagation reducing computational time.

Performance Metrics for COP and VOP for PID controller

Table S11: Performance metrics for controller including rise time (t_r_), steady-state temperature (T_ss_), standard deviation (T_ss,σ_), percentage overshoot (OS) and settling time (t_s_).

| **Description** | **COP** | | | **VOP** | | | |  |
| --- | --- | --- | --- | --- | --- | --- | --- | --- |
|  | **L1** | **L2** | **L3** | **L1** |  | **L2** | **L3** |  |
| t_r_ min  T_SS_± T_ss,σ_ °C  OS %  t_s_ min | 0.32 | - | 0.65 | 0.58 |  | - | 1.27 |  |
|  | 59.99 ± 0.01 | - | 60.00 ± 0.00 | 59.98 ± 0.01 |  | - | 59.95 ± 0.03 |  |
|  | - | - | 0.39 | - |  | - | - |  |
|  | 0.33 | - | 0.65 | 0.73 |  | - | 1.25 |  |

**References:**

1. Fuentes D, Oden JT, Diller KR, et al. Computational Modeling and Real-Time Control of Patient-Specific Laser Treatment of Cancer. *Annals of Biomedical Engineering*. 2009;37(4):763-782. doi:https://doi.org/10.1007/s10439-008-9631-8 .
2. Welch AJ, Martin. *Optical-Thermal Response of Laser-Irradiated Tissue*. Springer Nature; 1995. doi:https://doi.org/10.1007/978-90-481-8831-4.
3. Fuentes D, Walker C, Elliott A, Shetty A, Hazle JD, Stafford RJ. Magnetic resonance temperature imaging validation of a bioheat transfer model for laser‐induced thermal therapy. International Journal of Hyperthermia. 2011;27(5):453-464. doi: <https://doi.org/10.3109/02656736.2011.557028>
4. Mohammadi A, Bianchi L, Asadi S, Saccomandi P. Measurement of Ex Vivo Liver, Brain and Pancreas Thermal Properties as Function of Temperature. *Sensors*. 2021;21(12):4236. doi: <https://doi.org/10.3390/s21124236>.
5. Vincelette RL, Curran MP, Danish SF, Grissom WA. Appearance and modeling of bubble artifacts in intracranial magnetic resonance-guided laser interstitial thermal therapy (MRg-LITT) temperature images. *Magnetic Resonance Imaging*. Published online April 2023. doi: <https://doi.org/10.1016/j.mri.2023.03.022>.
6. Lad Y, Avesh Jangam, Carlton H, et al. Development of a Treatment Planning Framework for Laser Interstitial Thermal Therapy (LITT). *Cancers*. 2023;15(18):4554-4554. doi:https://doi.org/10.3390/cancers15184554.
7. “Density» IT’IS Foundation.” https://itis.swiss/virtual-population/tissue-properties/database/density/ Accessed on 03/12/2025.
8. “Heat capacity » IT’IS foundation.” <https://itis.swiss/virtual-population/tissue-properties/database/heat-capacity/> Accessed on 03/12/2025.
9. “Thermal conductivity » IT’IS foundation.” <https://itis.swiss/virtual-population/tissue-properties/database/thermal-conductivity/> Accessed on 03/12/2025.
10. G. Schooneveldt et al., “Hyperthermia treatment planning including convective flow in cerebrospinal fluid for brain tumour hyperthermia treatment using a novel dedicated paediatric brain applicator,” Cancers, vol. 11, no. 8, p. 1183, Aug. 2019, doi: 10.3390/cancers11081183.
11. Final Advanced Materials, “Pure Silica Fibre”, <https://www.final-materials.com/gb/24-pure-silica-fibre>. Accessed on 03/12/2025.
12. Namakshenas P, Di Matteo FM, Bianchi L, et al. Optimization of laser dosimetry based on patient-specific anatomical models for the ablation of pancreatic ductal adenocarcinoma tumor. *Scientific Reports*. 2023;13(1). doi: <https://doi.org/10.1038/s41598-023-37859-7>
13. Yaroslavsky AN, Schulze PC, Yaroslavsky IV, Schober R, Ulrich F, Schwarzmaier H-J. Optical properties of selected native and coagulated human brain tissues in vitro in the visible and near infrared spectral range. *Physics in Medicine and Biology*. 2002;47(12):2059-2073. doi: <https://doi.org/10.1088/0031-9155/47/12/305> .
14. Kim BM, Jacques SL, Rastegar S, Thomsen S, Motamedi M. Nonlinear finite-element analysis of the role of dynamic changes in blood perfusion and optical properties in laser coagulation of tissue. *IEEE Journal of Selected Topics in Quantum Electronics*. 1996;2(4):922-933. doi: <https://doi.org/10.1109/2944.577317>
15. Lim CK, Chung BJ. Natural convection experiments on the upward and downward faces of inclined plates using an electroplating system. *Heat and Mass Transfer*. 2014;51(5):713-722. doi:https://doi.org/10.1007/s00231-014-1450-x.
16. Nellis GF, Klein SA. *Introduction to Engineering Heat Transfer*.; 2020. doi:https://doi.org/10.1017/9781316832226.
17. “Medtronic Visualase™ MRI-Guided Laser Ablation.’’ <https://www.medtronic.com/content/dam/medtronic-com/products/neurological/laser-ablation/documents/visualase-brochure.pdf> Accessed on 03/07/2025
18. He X, McGee SM, Coad JE, et al. Investigation of the thermal and tissue injury behaviour in microwave thermal therapy using a porcine kidney model. 2004;20(6):567-593. doi:https://doi.org/10.1080/0265673042000209770.
19. Moritz AR, Henriques FC. Studies of Thermal Injury: II. The Relative Importance of Time and Surface Temperature in the Causation of Cutaneous Burns. Am J Pathol. 1947;23(5):695-720.
20. Schutt DJ, Haemmerich D. Effects of variation in perfusion rates and of perfusion models in computational models of radio frequency tumor ablation. *Medical Physics*. 2008;35(8):3462-3470. doi:https://doi.org/10.1118/1.2948388.
21. Sri Kamal Kandala, Liapi E, Whitcomb LL, Anilchandra Attaluri, Ivkov R. Temperature-controlled power modulation compensates for heterogeneous nanoparticle distributions: a computational optimization analysis for magnetic hyperthermia. *International Journal of Hyperthermia*. 2018;36(1):115-129. doi:https://doi.org/10.1080/02656736.2018.1538538.
22. Nelson DA, Nunneley SA. Brain temperature and limits on transcranial cooling in humans: quantitative modeling results. *European Journal of Applied Physiology*. 1998;78(4):353-359. doi:https://doi.org/10.1007/s004210050431.
23. Gentilal N, Miranda PC. Heat transfer during TTFields treatment: Influence of the uncertainty of the electric and thermal parameters on the predicted temperature distribution. *Computer Methods and Programs in Biomedicine*. 2020;196:105706. doi:https://doi.org/10.1016/j.cmpb.2020.105706.
24. Marissa Nichole Rylander, Feng Y, Zhang Y, et al. Optimizing heat shock protein expression induced by prostate cancer laser therapy through predictive computational models. *Journal of Biomedical Optics*. 2006;11(4):041113-041113. doi: <https://doi.org/10.1117/1.2241310> .
25. Qin Z, Balasubramanian SK, Wolkers WF, Pearce JA, Bischof JC. Correlated Parameter Fit of Arrhenius Model for Thermal Denaturation of Proteins and Cells. *Annals of biomedical engineering*. 2014;42(12):2392-2404. doi:https://doi.org/10.1007/s10439-014-1100-y.
26. COMSOL Multiphysics Livelink for Simulink. Available online: <https://doc.comsol.com/5.6/doc/com.comsol.help.llsimulink/LiveLinkForSimulinkUsersGuide.pdf> (accessed on 10/30/2024).
27. Carslaw, H. S., & Jaeger, J. C. (1959). Conduction of heat in solids, Clarendon.
28. Fuentes D, Yusheng Feng, Elliott A, et al. Adaptive Real-Time Bioheat Transfer Models for Computer-Driven MR-Guided Laser Induced Thermal Therapy. *IEEE Transactions on Biomedical Engineering*. 2010;57(5):1024-1030. doi: <https://doi.org/10.1109/tbme.2009.2037733>.
29. Desclides M, Ozenne V, Bour P, et al. Real-time automatic temperature regulation during in vivo MRI-guided laser-induced thermotherapy (MR-LITT). *Scientific Reports*. 2023;13(1). doi: <https://doi.org/10.1038/s41598-023-29818-z>.
